# Omics-Facilitated Crop Improvement for Climate Resilience and Superior Nutritive Value

**DOI:** 10.3389/fpls.2021.774994

**Published:** 2021-12-01

**Authors:** Tinashe Zenda, Songtao Liu, Anyi Dong, Jiao Li, Yafei Wang, Xinyue Liu, Nan Wang, Huijun Duan

**Affiliations:** ^1^State Key Laboratory of North China Crop Improvement and Regulation, Hebei Agricultural University, Baoding, China; ^2^Department of Crop Genetics and Breeding, College of Agronomy, Hebei Agricultural University, Baoding, China; ^3^Department of Crop Science, Faculty of Agriculture and Environmental Science, Bindura University of Science Education, Bindura, Zimbabwe; ^4^Academy of Agriculture and Forestry Sciences, Hebei North University, Zhangjiakou, China

**Keywords:** abiotic stress, biotic stress, pan-genomes, nutritive traits, multi-omics technologies, systems biology approach, genomics assisted breeding (GAB), single cell transcriptomics

## Abstract

Novel crop improvement approaches, including those that facilitate for the exploitation of crop wild relatives and underutilized species harboring the much-needed natural allelic variation are indispensable if we are to develop climate-smart crops with enhanced abiotic and biotic stress tolerance, higher nutritive value, and superior traits of agronomic importance. Top among these approaches are the “omics” technologies, including genomics, transcriptomics, proteomics, metabolomics, phenomics, and their integration, whose deployment has been vital in revealing several key genes, proteins and metabolic pathways underlying numerous traits of agronomic importance, and aiding marker-assisted breeding in major crop species. Here, citing several relevant examples, we appraise our understanding on the recent developments in omics technologies and how they are driving our quest to breed climate resilient crops. Large-scale genome resequencing, pan-genomes and genome-wide association studies are aiding the identification and analysis of species-level genome variations, whilst RNA-sequencing driven transcriptomics has provided unprecedented opportunities for conducting crop abiotic and biotic stress response studies. Meanwhile, single cell transcriptomics is slowly becoming an indispensable tool for decoding cell-specific stress responses, although several technical and experimental design challenges still need to be resolved. Additionally, the refinement of the conventional techniques and advent of modern, high-resolution proteomics technologies necessitated a gradual shift from the general descriptive studies of plant protein abundances to large scale analysis of protein-metabolite interactions. Especially, metabolomics is currently receiving special attention, owing to the role metabolites play as metabolic intermediates and close links to the phenotypic expression. Further, high throughput phenomics applications are driving the targeting of new research domains such as root system architecture analysis, and exploration of plant root-associated microbes for improved crop health and climate resilience. Overall, coupling these multi-omics technologies to modern plant breeding and genetic engineering methods ensures an all-encompassing approach to developing nutritionally-rich and climate-smart crops whose productivity can sustainably and sufficiently meet the current and future food, nutrition and energy demands.

## Introduction

Optimizing climate-change adaptation, agricultural productivity, food security and environmental protection is the grand challenge confronting scientists in this 21st century. The unequivocal change in climate, manifested in form of elevated average temperatures, global warming, sporadic and unreliable rainfalls, and enlargement of affected terrestrial regions under flood or water deficit is contributing to the expansion of drought or salinity-prone regions that are characterized by diminished plant growth and crop productivity (Lamaoui et al., 2018). Additionally, climate related changes will likely boost up the severity of both sole and combined abiotic stresses, especially drought, heat, salinity, cold, and submergence (Pandey et al., 2017; Anwar et al., 2021). Moreover, these climate change scenarios harshen the biotic stresses by boosting up the insect, pests or pathogen numbers and disease severity, stimulating weed species proliferation, dwindling soil beneficial microbes, and threatening vital plant pollinators (Kole et al., 2015; Raza A. et al., 2019; Shahzad et al., 2021). These effects have far-reaching implications for global food security, by significantly impacting plant growth, development and productivity, and consequently, global agricultural production (Dhankher and Foyer, 2018; Nhamo et al., 2019). This is occurring against the backdrop of a continued spiraling of world human population, spurred by relatively high levels of fertility in developing countries (UN, 2017), with modest projections pointing to 9.15 billion people by the year 2050 (Alexandratos and Bruinsma, 2012). This is exacerbating pressure on the agricultural production and food supply systems, since 56% more food will need to be produced to feed additional 3 billion mouths using the same or less quantity of resources as compared to the year 2010 (Ranganathan et al., 2018). More worryingly, around 800 million and 2 billion people are already facing acute food shortages and malnutrition problems, respectively, as access to nutritious foods is out of reach of many (FAO, 2019; Fiaz et al., 2021). Further, the edaphic environment, upon which our agricultural system relies for sustenance and provision of food to humans, is facing serious challenges related to natural resource degradation and decline as well as biodiversity erosion (Wassie, 2020; Zandalinas et al., 2021).

Given the scenario highlighted above, innovative sustainable crop production efforts are required to ensure optimized resilience under climate change conditions (Vaughan et al., 2018). Developing climate resilient crops, increasing efficiency of natural resource use, linking agricultural intensification with natural ecosystem protection, and diversification of agricultural systems have been widely proposed as sustainable solutions to address these challenges (Gil et al., 2017; Dhankher and Foyer, 2018; Evans and Lawson, 2020). These strategies will facilitate the closing of three main types of gaps, viz., the food gap, land gap, and greenhouse gases (GHG) mitigation gap (for detailed explanations, see The World Resources Institute, 2019). In particular, development of climate resilient crop cultivars with desired agronomic traits has been advocated as the most plausible, economical, sustainable and efficient way to adapt our agricultural system to climate change (Mba et al., 2012; Kumari et al., 2020; Kim J.H. et al., 2021). Breeding for climate smart crop cultivars will entail exploring crop wild relatives and revisiting neglected and underutilized species for the untapped novel allelic variation harbored by those species, thereby broadening the genetic variation available for crop breeders’ use (Brozynska et al., 2016; Gupta et al., 2017; Ananda et al., 2020; Kilian et al., 2020; Kamenya et al., 2021). Additionally, there will be need to employ advanced crop breeding techniques and methodologies, integrated with conventional and improved data analysis pipelines (Ahmar et al., 2020; Bohra et al., 2020; Pourkheirandish et al., 2020; Qaim, 2020; Steinwand and Ronald, 2020).

Fortunately, the flourishing developments in omics technologies have revolutionized our crop improvement endeavors, by fortifying crop breeders’ toolboxes and galvanizing omics-assisted breeding programs targeting various agronomic traits (Langridge and Fleury, 2011; Li and Yan, 2020). Omics technology is a modern molecular tool useful in understanding functional genomic systems in an organism (Hu et al., 2018; Banerjee et al., 2019), and involves DNA sequencing and profiling of the expressed transcripts and translated proteins (Missanga et al., 2021). With the term “omics” being a derivative of the Greek word “-ome” meaning “whole,” omics refer to scientific disciplines that study different types of biological molecules constituting complete biological systems (SETAC, 2019). These disciplines encompass genomics, transcriptomics, proteomics, metabolomics, and phenomics (Hasin et al., 2017; Khalid et al., 2019).

Specifically, recent advances in genome sequencing techniques, coupled with omics-platforms generated data, have facilitated the availability of enormous genomic and transcriptomic data for various crop species, and have significantly improved gene discovery, gene expression profiling, marker-assisted selection, domestication of underutilized species, and introgression of unique and key traits into desired crops (Pathak et al., 2018; Muthamilarasan et al., 2019; Cortés and López-Hernández, 2021). This is now permitting us to routinely delineate the molecular and genetic underpinnings to the several phenotypic traits of agricultural importance (Scossa et al., 2021). Integrated with other modern crop improvement strategies such as speed breeding and gene editing technologies, omics approaches now facilitate rapid creation of elite climate smart cultivars with desired traits such as enhanced productivity, abiotic and biotic tolerance, and nutritive quality (Gao, 2021; Kumar R. et al., 2021; Singh R. K. et al., 2021).

Here, citing some relevant examples, we appraise our knowledge on the recent progress in omics approaches and how these developments, integrated with other modern plant breeding, data analysis, and gene editing technologies, are altering the crop improvement landscape related to abiotic and biotic stress tolerance, higher nutritional quality and other key agronomic traits, thereby facilitating global food and nutrition security.

## Omics Approaches for Crop Improvement: an Overview

In modern molecular biology, the suffix “-omics” specially refers to a collection of technologies applied to the analysis of a huge and complete data set of a particular class or type of biological molecule in a cell, tissue, organ, or whole organism (Zaitlin, 2020). In other words, plant molecular biology revolves around investigating cellular processes, their genetic determinants, and interactions with environmental alterations, and such a multi-dimensional and comprehensive inquiry involves large-scale experiments targeting entire genetic, structural, or functional components. These large scale studies are what are known as “omics” (Deshmukh et al., 2014). The omics sub-disciplines at the forefront of fundamental systems biology studies and contemporary crop improvement interventions are genomics, transcriptomics, proteomics, metabolomics, and phenomics (Hasin et al., 2017; Dubey et al., 2019a); which chiefly involve comprehensive investigation of the genome, transcriptome, proteome, metabolome, and phenotypes, respectively (Table 1). All of these omics branches are closely linked to bioinformatics (Zaitlin, 2020).

**TABLE 1:** An overview of main omics strategies for crop improvement.

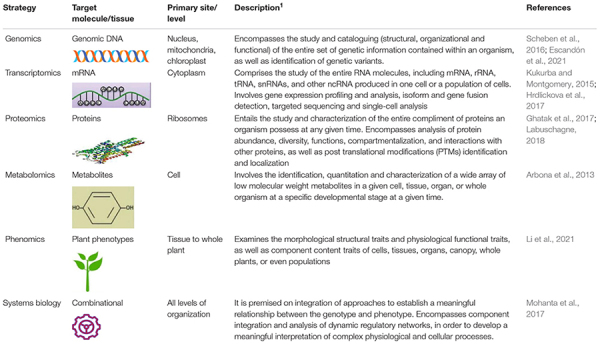

*^1^mRNA, messenger RNA; rRNA, ribosomal RNA; tRNA, transfer RNA; snRNA; ncRNA, non-coding RNA.*

**FIGURE 1:**
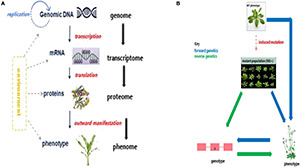
Link among the major biological molecules and genetic approaches for crop improvement. **(A)** A cascade of interactions among the major biological molecules constituting the central dogma of molecular biology. Owing to the complexity of biological systems and molecular interactions, the simplistic arrows shown here offer only a general scheme of cascading influence. Dotted lines imply that the environment affects the biomolecules at different levels. **(B)** Genetic approaches for crop improvement. The canonical forward genetic approach involves creating variation (either naturally or via induced mutations) in a population; identifying interesting and novel phenotypes; and then cloning the gene/s responsible for the identified phenotypic variation. Reverse genetic approach involves first carrying out genotypic screening of the mutant population to identify novel induced mutations in candidate genes, and then perform phenotypic evaluation of those individuals harboring putative mutations (Jankowicz-Cieslak and Till, 2015; SETAC, 2019).

In general, the analyses of the -omics fields are modeled along the structure of Francis Crick‘s (1954) classical central dogma of molecular biology (through targeted investigation of each molecule at a particular level). Put simply, the genome, transcriptome, proteome, metabolome, and phenome constitute different layers of the omics cascade, each of which defines a biosystem or an organism at different biomolecular levels (Jendoubi, 2021; Figure 1A). However, the complexity of biological systems means that dynamic environmental and spatio-temporal molecular interactions do not actually follow this simple path of reductionism and cannot be studied from the static topology point of view (Franklin and Vondriska, 2011; Wolkenhauer and Muir, 2011). Hence, a systems biology approach provides a holistic way for dissecting the underlying genetic and molecular mechanisms governing specific traits of economic importance (Pazhamala et al., 2021). The advent of omics strategies, coupled with other technological inventions such as gene sequencing and mutagenesis, has offered new dimensions in crop improvement programs, by facilitating improved gene function prediction, and better dissection of molecular mechanisms underlying important agronomic traits (Kumar R. et al., 2021). This is essential for the development of superior crop cultivars enhanced with greater yield, stability, abiotic and biotic stress tolerance, and nutritional composition, through introgressing genes or QTL from identified donor genotypes, either via forward genetics or reverse genetic approaches (Figure 1B; Bahuguna et al., 2018).

## Genomics and Pan-Genomics

### High Quality Reference Genomes as Vital Resources for Accurate Annotation of Gene Structure, Content and Variation

Recent cost reductions in high throughput (HTP) sequencing and rapid improvements in sequence assembly algorithms and surveying platforms have facilitated for the readily availability of genomic tools and resources for several crops (Bohra, 2013; Jayakodi et al., 2021). These tools include high quality reference genomes, DNA markers, and genetic maps, which are essential for functional and comparative genomic studies, as well as molecular crop improvement (Zhang and Hao, 2020). Especially, the availability of reference genomes for several major crops and the ability to perform HTP resequencing have enabled us to demarcate genes and other regulatory sequences, map genomic variations, refine gene models and better understand gene functions (Morrell et al., 2012; Schreiber et al., 2018; Zhang Q. et al., 2020). Researchers can now routinely perform genome-wide scans for genes controlling key traits of agronomic importance in crops (Zhang et al., 2019; Nakano and Kobayashi, 2020).

Genome sequencing technologies have evolved from the classical Senger method (first generation), through next generation sequencing (NGS), to third generation sequencing (TGS) approaches. For detailed reviews on these sequencing approaches, we refer you to previous papers (Li et al., 2018; Cui et al., 2020). Through these technologies, especially NGS and TGS, several crop genomes have been sequenced (Purugganan and Jackson, 2021), including those for soybean (*Glycine max* L., Shen et al., 2018), lablab (*Lablab purpureus* L. Sweet) and other major grain legumes (see Varshney et al., 2015; Missanga et al., 2021), ten top most world food crops (see Varshney et al., 2021), major and minor millets (see Vetriventhan et al., 2020; Singh R. K. et al., 2021), several cereal crops including orphaned species (see Table 1 of our most recent paper, Zenda et al., 2021), diverse crop species (Michael and Jackson, 2013; see Bevan and Uauy, 2013; Bevan et al., 2017; Mohanta et al., 2017; Schreiber et al., 2018), and fruit crops (Li et al., 2019). Among these sequenced crop species are crop wild relatives and underutilized species (Chang et al., 2019; Vetriventhan et al., 2020), which have been recognized as excellent sources of novel genetic diversity for future crop improvements (Schreiber et al., 2018; Singh R. K. et al., 2021). Thus, complete genome assemblies for hundreds of crop species are now available in public repositories [Kersey, 2019; Pazhamala et al., 2021; Sequenced plant genomes – CoGepedia (genomevolution.org)] and several genome databases and tools have been created (for extensive review, see Bohra, 2013; Chen et al., 2018; Varshney et al., 2021). Additionally, progress in genome sequencing and HTP genotyping has opened a window for increased *de novo* domestication of crop wild relatives and orphan species for accelerated crop improvement for abiotic stress and higher nutritive value (Morrell et al., 2012; Bevan and Uauy, 2013; Bohra et al., 2014; Schreiber et al., 2018; Gasparini et al., 2021). Taken together, the recent fast-paced developments in genome sequencing and assembly are enabling easy decoding of intricate crop genomes for genes and alleles controlling key agronomic traits.

### Large-Scale Resequencing and Pan-Genomes Facilitating Identification and Analysis of Species-Level Genomic Variations

Genetic diversity among species and within populations is the mainstay of crop improvement and genetic dissection of complex traits (Gao, 2021). In plant genomes, natural variations emanate from single nucleotide polymorphisms (SNPs), small insertions and deletitions (InDels, <50 nucleotides), and structural variants (SVs, >50 nucleotides) (Vishwakarma et al., 2017). Large polymorphisms, encompassing large-scale duplications, presence/absence variants (PAVs), copy number variants (CNVs), deletions and rearrangements constitute the SVs (Saxena et al., 2014; Ho et al., 2020; Zhao et al., 2020). Particularly, SVs have been recognized as important sources of functionally consequential genetic variations within species (Tao et al., 2019), and have significantly contributed to crop domestication, evolution and improvement (Qin et al., 2021). Owing to developments in high quality genome sequencing and resequencing, an increasing number of crop genomic studies based on high quality assemblies have resolved SVs and facilitated the accurate annotation of functional gene variants among selected accessions (Table 2; Alonge et al., 2020; Qin et al., 2021).

**TABLE 2:** Examples of pan-genome studies conducted in major crops and related species.

**Crop species**	**Chr. No. and ploidy level**	**Approach used for pan-genome construction**	**No. of accessions**	**Sequencing strategy**	**Pan-genome**				**References**
					**No. of pan-genes**	**Core genes (%)**	**Variable genes (%)**	**Gene variants**	
*Zea mays*	2n = 2x = 20 Diploidized tetraploid	Pan-transcriptomics	503	Illumina Hiseq	41,903	39.12	60.88	∼1.628 million SNPs	Hirsch et al., 2014
*Oryza sativa*	2n = 2x = 24 Diploid	*De novo* assembly	3	Illumina HiSeq	40,362	92.17	7.83	–	Schatz et al., 2014
*Oryza sativa*, O. *rufipogon*	2n = 2x = 24 Diploid	*De novo* assembly	66	Illumina HiSeq	42,580	61.94	38.06	23 million sequence variants, comprising SNPs and 10,872 gene PAVs	Zhao Q. et al., 2018
*Oryza sativa*	2n = 2x = 24 Diploid	Map-to-pan	3010	Illumina HiSeq, PacBio	48,098	48.5–58.3	41.7–51.5	29 million SNPs, 2.4 million small inDels, 93,683 SVs, high number of PAVs	Wang W. et al., 2018
*Triticum aestivum*	2n = 6x = 42 (AABBDD) allopolyploid	Iterative mapping and assembly	18	Illumina HiSeq	140,500	57.70	42.30	36.4 million SNPs,	Montenegro, 2017
*Glycine max*	2n = 2x = 40 Diploidized polyploid	Graph based *de novo* assembly	27	PacBio, Illumina HiSeq	57,492	50.1	49.9	31.87 million SNPs; 723, 862 PAVs; 27,531 CNVs; 21,886 TLEs; 3,120 IEs	Liu Y. et al., 2020
*Glycine soja*	2n = 2x = 40 Diploidized polyploid	Sequencing and *de novo* assembly	7	Illumina HiSeq 2000	59,080	48.60	51.40	∼ 25.41–33.04 million SNPs, 338 PAVs, 1978 CNVs	Li et al., 2014
*Brassica napus*	2n = 2x = 38 (AACC) alloptetraploid	Sequencing and *de novo* assembly, PAV based.	8	PacBio, Illumina paired-end short read, Hi-C technologies	152,185	∼56	44	16,720 PAVs, 1,360 inversions, 3,716 translocations, millions of SNPs and InDels	Song et al., 2020
*B. oleracea*	Diploid	Iterative mapping and assembly	9	Illumina	61,379	81.29	18.71	4,815 million SNPs, and high number of PAVs	Golicz et al., 2016b
*Brassica rapa* and *B. oleracea*	2n = 2x = 10 *B. rapa*, A genome); 2n = 2x = 9 (*B. oleracea*, C genome)	Whole genome resequencing	318	Illumina HiSeq 2000	–	–	–	2.249 million and 3.852 million SNPs; 303,617 and 417,004 InDels for *B. rapa* and 119 *B. oleracea*, respectively.	Xu et al., 2012
*Capsicum annuum; C. baccatum, C. chinense, C. frutescens*	2n = 2x = 24 Diploid	Iterative mapping and assembly	383	Illumina HiSeq	51,757 high quality	55.7	44.3	Numbers not specified	Qu et al., 2018
*Lycopersicum esculentum*	2n = 2x = 24 Diploid	*De novo* assembly	725	Illumina NextSeq	40,283	74.2	25.8		Gao et al., 2019
*Helianthus annuus*	2n = 2x = 34 Diploid	Map-to-pan	493	Illumina Hiseq	61,205	73	27		Hübner et al., 2019
*Arabidopsis thaliana*	2n = 2x = 10 Haploid	Comparative *de novo* assembly	18	Illumina HiSeq2000	37,789	69.7	30.3	–	Contreras-Moreira et al., 2017
*Hordeum vulgare*	2n = 2x = 14	Assembly comparison and CSCS	20	Illumina RNA-Seq, PacBio Iso-Seq	40,176 OGGs	54.74	45.26	1.586 million PAVs	Jayakodi et al., 2020
*Sorghum bicolor*	2n = 2x = 20 Diploid	Iterative mapping and assembly	176	Illumina RNA-Seq	35,719	47.09	52.91	2 million SNPs and inDels, 983,060 CNVs,	Ruperao et al., 2021
*Sesamum indicum*	2n = 2x = 26	Whole genome alignment	5	–	26,472	58.21	41.79	–	Yu J. et al., 2019
*Sorghum bicolor* ssp. ^1^	2n = 2x = 20	*Denovo* assembly	16	Illumina short reads & PacBio long reads	44,079	36	64	15.293 million SNPs, 0.3–1.5 million inDels per genome, 429-1118 CNVs per genome, 19,359-147,899 PAVs per genome	Tao et al., 2021

*^1^*S. bicolor* ssp., comprises *Sorghum bicolor* and its progenitors *Sorghum bicolor* ssp. *verticilliflorum*, *S. bicolor spp. propinquum*, *S. bicolor spp. drummondii*, *S. bicolor spp. bicolor* CSCS, clustering of single-copy sequences; OGGs, orthologus gene groups; SNPs, single nucleotide polymorphisms; CNVs, gene copy number variations; PAVs, presence/absence variations; TLEs, translocation events; IEs, inversion events.*

Genome SVs can be detected using any of the three approaches, viz., *de novo* domestication, resequencing, and pan-genome (Saxena et al., 2014). Particularly, *de novo* assembly of multiple high-quality reference genome sequences and their subsequent comparison by pair-wise sequence alignment has proved a very powerful and accurate method of detecting all types of SVs at base-level resolution (Jayakodi et al., 2021). For example Li et al. (2014) constructed a *de novo* assembly-based pan-genome of *Glycine soja*, the wild relative of cultivated soybean *Glycine max*, by sequencing seven phylogenetically linked accessions and observed lineage-specific genes and CNV-possessing genes by intergenomic comparisons, with some CNV-containing genes exhibiting evidence of positive selection and linked to variation of key agronomic traits such as anthesis and maturity time, seed composition, final biomass, and biotic resistance. Additionally, they identified that 80% of the *Glycine soja* pan-genome constituted the core genome, whereas 20% (the dispensable genome) showed greater variation than the core genome, probably reflecting the dispensable genome‘s role in acclimation to diverse environments (Li et al., 2014).

Large-scale resequencing of diverse crop germplasm and genome-wide association studies (GWAS) are laying bare the extent of genome variation, the genetic architecture, and link between the phenotype and genotype, which are gateways in deciphering the genes underpinning several agronomically important traits in various crops (Huang and Han, 2014; Xu and Bai, 2015; Zhang Q. et al., 2020; Ye et al., 2021). Some of the major crops that have been resequenced include sorghum (McCormick et al., 2018; Cooper et al., 2019), maize (Lai et al., 2010; Xu et al., 2014), soybean (Zhou et al., 2015), tomato (*Solanum lycopersicum* L., Roohanitaziani et al., 2020; Ye et al., 2021), eggplant (*Solanum melongena* L.) and its wild relative (*Solanum incanum* L.) (Gramazio et al., 2019), rice and its wild progenitors (*Oryza rufipogon* L. and *Oryza nivara* L.) (Xu et al., 2012), *Brassica rapa* L. and *Brassica. oleracea* L. (Cheng et al., 2016), and several crop species (reviewed in Varshney et al., 2021). The TGS approaches such as PacBio Single Molecule Real Time, Illumina Tru-seq Synthetic Long-Read and Oxford Nanopore technologies employ the use of single molecule reads (see Li et al., 2018; Cui et al., 2020 for extensive review), which can exceed megabases in length, thereby providing unprecedented opportunities to resolve SVs missed by short read approaches (Schreiber et al., 2018; Michael and VanBuren, 2020). For example, Zhou et al. (2015) resequenced 302 soybean accessions (comprising wild, landraces, and improved cultivars) at > 11 × depth and then performed GWAS analysis of these accessions‘ sequences, which identified 13 previously uncharacterized loci for key agronomic traits including plant height and oil content, among others. As the costs for DNA sequencing continue to decline and new innovations in gene editing, machine learning and data algorithms gather pace, whole genome resequencing approaches will not only help in better understanding of the genetic basis of complex traits, but will increasingly play important roles in QTL mapping and gene identification, consequently accelerating crop improvement for climate resilience and higher nutritive value via genomics assisted breeding (GAB).

The concept of pan-genomes has been propelled by the realization that a single reference genome sequence is insufficient to represent the full spectrum of genetic variation occurring within a species (Golicz et al., 2016a; Bayer et al., 2020). Pan-genome involves the non-redundant assemblage of genes and/or DNA sequences in a clade or a species (Lei et al., 2021), and encompasses core genome (containing genes present all accessions) and variable genome (comprising partially shared and accession specific genes) (Saxena et al., 2014; Tahir ul Qamar et al., 2020). Since a pan-genome provides an entire complement of genomic diversity repertoire of a genus, pan-genome analysis is a more robust, comprehensive and indispensable approach to identify gene content variation and perform a whole-species genetic diversity analysis (Tao et al., 2019; Khan et al., 2020).

Crucially, pan-genomes usually contain within-species CNVs and PAVs (Scheben et al., 2016), and such SVs have been observed to influence traits of agronomic importance in crops (Zuo et al., 2015; Tao et al., 2019; Khan et al., 2020). Notably, variable gene annotations often exhibit similarities across plant species, with genes for biotic and abiotic stress tolerance frequently enriched within variable gene clusters (Bayer et al., 2020). It is no surprising that pan-genomics is a hot topic at the present moment, with pan-genomic studies facilitating the dissection of the genetic variation, which is critical for linking the desirable phenotypes to major agronomical traits (Danilevicz et al., 2020; Coletta et al., 2021). Ever since the concept of pan-genomes was first established in 2005 by Tettelin et al. (2005), several crop pan-genomes have been developed, including for maize, soybean and wheat among others (Table 2).

Since pan-genomes can reveal the extent of novel alleles and genes in crop wild relatives, the novel candidate genes that may be linked to adaptation to numerous biotic and abiotic stresses can be introgressed into cultivated crops to increase their resilience to climate variability. Essentially, genes harboring SVs and large-effect mutations showing association with important agronomic phenotypes (as inferred by mapped QTLs) can be harnessed to develop molecular markers for the SV containing regions and test new allelic combinations (Li et al., 2014), thereby providing new resources for designing new crop cultivars (Khan et al., 2020).

Already (Zenda et al., 2021), we have highlighted that transposable elements (TEs), which are ubiquitous mobile DNA sequences with the propensity to transverse along the genome (Makalowski et al., 2019), are becoming a new research avenue for crop genome analysis and helping us better understand crop abiotic and biotic stress responses. TE transposition has been shown to modulate transcriptional activity of contiguous genes through regulation of epigenomic profile of the region (Ariel and Manavella, 2021). Additionally, TEs largely contribute to genome size variation (Dubin et al., 2018; Anderson et al., 2019) and SVs among different crop species (Tao et al., 2019; Coletta et al., 2021). Particularly, TEs have been shown to activate important gene allelic or regulatory variation in abiotic stress responses (Makarevitch et al., 2015). As the omics technology develop, new methodologies for comprehensive TE annotation and analysis will also need to keep pace with these developments, in order to help us better decipher how TEs regulate plant phenotypic responses to abiotic stresses (for a detailed review, see Zenda et al., 2021).

### Genetic Diversity Analysis and Mapping of Quantitative Traits

Dissecting the genetic basis of important agronomic traits, such as grain yield, grain size, flowering time, fiber quality and disease resistance is essential for manipulating and precise introgression of these traits in breeding programs (Würschum et al., 2012; Noble et al., 2018; Mérida-García et al., 2019; Shi Y. et al., 2019; Goddard et al., 2020). In other words, GAB is facilitated by the identification of molecular genomic markers linked to QTLs or genes underlying agronomic traits of interest, which are then utilized as useful tools for molecular breeding (Singh R.K. et al., 2020; Sinha et al., 2021). To that end, several GAB approaches have been deployed in various crop improvement programs, including marker-assisted backcrossing (MABC) to enhance β-carotene content in maize (Qutub et al., 2021); marker-assisted recurrent selection (MARS) to improve crown rot (*Fusarium pseudograminearum*) resistance in bread wheat (Rahman et al., 2020) and pod shattering resistance in soybean (Kim et al., 2020); as well as genomic selection (GS) to improve rice blast (*Magnaporthe oryzae*) resistance (Huang et al., 2019) and maize drought tolerance (Shikha et al., 2017). Meanwhile, molecular marker based applications such as gene linkage and quantitative trait loci (QTL) mapping have become more feasible owing to the recent advances in genotyping platforms and statistical genomics (Kulwal, 2018). More significantly, cost-effective NGS technologies have accelerated the development of molecular markers and their deployment in genetic diversity and phylogenic relationship analyses in various species. Molecular markers have been widely used to ascertain the magnitude of genetic diversity in cultivated and wild crop gene pools (see Kumar J. et al., 2021). Additionally, numerous studies have been performed to identify several QTLs for diverse traits of agronomic value in different crop species (see Nepolean et al., 2018; Choudhary et al., 2019; Kumar J. et al., 2019; Singh R.K. et al., 2020; Liu and Qin, 2021). For example, nine QTLs for grain yield under low soil nitrogen environments in maize (Ribeiro et al., 2018), major QTLs controlling grain yield under drought in pearl millet (Bidinger et al., 2007; Debieu et al., 2018), QTLs for plant height and flowering time in soybean (Cao et al., 2017), QTLs and candidate genes for root-knot nematode resistance in cowpea (*Vigna unguiculata* L.) (Santos et al., 2018), QTLs for Fusarium head blight resistance in barley (Huang et al., 2018), novel QTLs for salinity tolerance in rice (Pundir et al., 2021), QTLs controlling protein and oil contents and oil quality in groundnut (Sarvamangala et al., 2011), and QTLs for seed Fe and Zn content in chickpea (Sab et al., 2020) were identified, among others.

Especially, sequence-based and genome-wide distributed high-density SNP markers have been successfully used to characterize cultivated varieties and landraces based on their geographical origin, and have been efficient in the identification of varied levels of genetic diversity among diverse genotypes in gene pools (Kumar J. et al., 2021). Additionally, SNP markers have been used to map QTLs/genes controlling the target traits of agronomic importance in different crops such as maize (Cui et al., 2015), lentil (Kumar J. et al., 2021), soybean (Lee et al., 2015), cotton (Sun et al., 2017, 2018; Majeed et al., 2019), groundnut (Liang et al., 2017; Han et al., 2018) and several crops (Mammadov et al., 2012). Notably, SNPs have greatly supported GWAS in delineating the slightest possible genome variations linked to plant phenotypic variations (Bohra et al., 2020). Thus, GWAS improves the mapping resolution for accurate location of allele/QTL/genes underlying key agronomic traits (Huang and Han, 2014; Pang et al., 2020). Unsurprising, large-scale GWAS has become a powerful tool for performing efficient genome-phenotype association analysis and identification of causative QTL/genes for key agronomic traits in diverse crop species (Sun et al., 2017; Jha et al., 2020; Berhe et al., 2021; Kaur et al., 2021; Sinha et al., 2021). For instance, using a natural population comprising 713 upland cotton accessions, Sun et al. (2018) discovered a total of 10 and 15 SNPs that were significantly associated with relative survival rate and salt tolerance level, respectively, among which two SNPs (i46598Gh and i47388Gh) on genomic region D09 were simultaneously linked with the two traits. A GWAS using a diverse panel of 206 genotypes identified genetic loci associated with Striga (*Striga hermonthica*) resistance genes in sorghum (Kavuluko et al., 2021). The study detected secondary cell wall modification genes for lignin biosynthesis genes, including PMT2 Methyltransferase at position S2_59157949, secondary wall *NAC TF 4* at S6_60968111 and *early nodulin 93* at S10_2576197. Additionally, they identified the Fasciclin-like arabinogalactan protein 11 that regulates plasticity and integrity of cell walls at position S9_5732771, as well as revealing the association of Striga resistance with the Ethylene-responsive transcription factor ERF113 at S4_50512606. ERF113 is a key regulator of both jasmonic acid (JA) and salicylic acid (SA) mediated defense pathways in plants (Kavuluko et al., 2021). GWAS to understand the genetic architecture of grain yield (GY) and flowering time under drought and heat stresses in a collection of 300 tropical and subtropical maize inbred lines using 381 165 genotyping-by-sequencing (GBS) SNPs revealed that 1549 SNPs were significantly associated with all the 12 trait-environment combinations, with 193, 95, and 405 candidate genes associated with GY, anthesis-silking interval (ASI), and anthesis date (AD), respectively (Yuan et al., 2019). In the haplotype-based association mapping analysis, 19 candidate genes were identified for the 12 trait-environment combinations, and 156 SNPs were in the genic region of these candidate genes. Notably, four candidate genes (*GRMZM2G329229*, *GRMZM2G313009*, *GRMZM2G043764*, *and GRMZM2G10 9651*) overlapped in both the GBS SNP-based and the haplotype-based association mapping analyses, with three of these genes being associated with AD evaluated under different conditions (Yuan et al., 2019).

In another study, a GWAS analysis using 195 peanut accessions subjected to GBS approach produced a total of 13 435 high-quality SNPs, including 93 non-overlapping peak SNPs that were significantly associated with four (yield per plant, hundred-pod weight, hundred-seed weight, and pod branch number per plant) of the studied yield-related traits (Wang J. et al., 2019). Among the 93 yield-related-trait-associated SNP peaks, 12 were found to be co-localized with the QTLs identified in earlier related QTL mapping studies and these 12 SNP peaks were only related to three traits and were almost all positioned on chromosomes Arahy.05 and Arahy.16. Remarkably, gene annotation of the 12 co-localized SNP peaks identified 36 candidate genes, among which one interesting gene *arahy.RI9HIF* was picked as prime target for further evaluation. The rice homolog of *arahy.RI9HIF* produces a protein that has been shown to improve rice yield when over-expressed. Therefore, further validation of the *arahy.RI9HIF* gene, and other candidate genes particularly harbored within the more confident co-localized genomic regions, may hold much promise for considerably enhancing peanut yield (Wang J. et al., 2019). Besides these examples, several recent papers have highlighted how GWAS, supported by SNPs, have been successfully deployed to detect genomic regions and candidate genes for various crop agronomic traits (Mammadov et al., 2012; Mousavi-Derazmahalleh et al., 2019; Alqudah et al., 2020; Pang et al., 2020).

In recent years, the increased use of GS in GAB has facilitated for quick crop improvement (Shamshad and Sharma, 2018). In GS, genome-wide high throughput markers (such as SNPs) that are in LD with QTLs are used to estimate their effects through optimum statistical models, before genomic estimated breeding values (GEBVs) are computed for each individual to select potential elite lines (Shamshad and Sharma, 2018; Mérida-García et al., 2019; Voss-Fels et al., 2019). Two population types are a pre-requisite in GS, viz., a training/reference population comprised of a cohort of individuals with both genotypic and phenotypic data and a testing/breeding population consisting of candidate breeding lines with genotypic data only (Dwivedi et al., 2020; Xu et al., 2020). The predicted GEBVs are then used for selection, excluding the need for further phenotyping (Srivastava et al., 2020; Zenda et al., 2021). Therefore, GS remarkably shortens the breeding cycle as compared to traditional breeding strategies (Bhat et al., 2016; Sinha et al., 2021). Thus, GS is an economical and viable alternative to MAS and phenotypic selection of quantitative traits (Shikha et al., 2017; Mérida-García et al., 2019). It enables crop breeders to explore and increase genetic gain per selection per unit breeding cycle, consequently enhancing speed and efficiency of breeding programs, thus, enabling the faster development of improved crop cultivars to cope with the climate change induced challenges (Spindel et al., 2015; Bhat et al., 2016; Voss-Fels et al., 2019). Moreover, GS is more superior to traditional MAS approach because it addresses the effect of small genes which cannot be captured by the traditional MAS (Heffner et al., 2009). Already, GS has shown great promise for predicting genotype performance and selection of complex traits such as disease resistance (Arruda et al., 2015; Huang et al., 2019) and drought tolerance (Shikha et al., 2017; Cerrudo et al., 2018).

In order to resolve some difficulties surrounding the use of QTL information in marker assisted breeding and gene candidate identification, especially regarding complex abiotic stress related traits, meta-QTL analysis approach has been advanced. Meta-QTL analysis compiles QTL data from diverse studies together on the same genetic linkage map for identification of precise QTL region (Deshmukh et al., 2014). For instance, using 34 different mapping populations encompassing 53 different parental accessions, Soriano et al. (2021) conducted a meta-QTL analysis on 45 traits in durum wheat, including quality and abiotic and biotic stress-related traits. A total of 368 QTL distributed on all 14 chromosomes of the genomes A and B were projected, among which 171 QTLs were related to quality-related traits, 127 to abiotic stress and 71 to biotic stress. Resultantly, 318 QTLs were grouped in 85 meta-QTL (mQTL), of which 15 mQTL were selected as the most promising for candidate gene selection (Soriano et al., 2021). These 15 most promising mQTLs were located on nine different chromosomes and showed co-localized QTLs for several grain traits. Interestingly, five mQTLs (2B.7, 4A.1, 7A.1, 7A.2 and 7A3) harbored genes associated grain weight and size (*TaGS2*-B1, *TaCwi*-A1, *TaTEF*-7A, *TaGASR7*-A1 and *TaTGW*-7A), and two genes affecting grain yield and quality (*TaSdr*-A1 and *TaALP*-4A – involved in preharvest sprouting tolerance) and were located in mQTL2A.4 and mQTL4A.5, respectively (Soriano et al., 2021). In another study, meta-QTL analysis was applied for a large set of phenotypic data obtained from nine inter-connected biparental RIL populations and seven environments in order to reveal the genetic control of yield-related traits and seed protein content in pea (Klein et al., 2020). A total of 89 QTL explaining a part of phenotypic variation were detected across the seven pea chromosomes. The meta-analysis of these QTL revealed 27 consensus or mQTLs, with each mQTL corresponding to one to 15 initial QTLs. Notably, most mQTLs were consistently detected in different environments, regardless of significant environmental and GxE effects (Klein et al., 2020). The study pinpointed several robust mQTLs of seed yield and seed protein content in pea and proposed some candidate genes, including *Psat5g299400*, a gene belonging to the AUX/IAA family putatively involved in early response to auxin (found located on mQTL1.5 region), and *Psat2g005160*, a gene encoding ADP-glucose pyrophosphorylase (found located on the locus AGPS2 on mQTL1.1 region) (Klein et al., 2020) and previously shown to be associated with seed size QTL in pea (Smith et al., 1989). Other meta-QTL studies carried out to identify mQTLs for various quantitative traits of agronomic importance in crops are available for soybean (Deshmukh et al., 2014), maize (Chen et al., 2017; Guo et al., 2018), barley (Zhang X. et al., 2017), wheat (Safdar et al., 2020), rice (Raza Q. et al., 2019; Selamat and Nadarajah, 2021), and cotton (Said et al., 2013), among others. The useful information generated from these mQTL studies facilitates the cloning and pyramiding of QTLs to create new crop cultivars with specific quantitative traits and speed up breeding programs via MAS.

Linkage mapping using artificially created segregating populations has been the most conventional method used to dissect the genetic basis of crop traits (Kulwal, 2018; Noble et al., 2018). Different genetic populations have been exploited to identify thousands of QTLs for several agronomic traits, especially recombinant inbred lines, because of their simple development, balanced parental mixture, repeated phenotyping, and relatively high mapping power (Liang et al., 2021a). Other mapping population types include introgression lines, advanced backcross populations, F2 populations, double-haploid populations, and backcross populations (reviewed in Kaur et al., 2021; Zenda et al., 2021).

However, association mapping (AM), based on linkage dis-equilibrium (LD) in natural population is a powerful and highly desirable approach in quickly and efficiently dissecting important traits in plants (Nachimuthu et al., 2015; Zhao et al., 2017). AM is a strategy that accounts for thousands of polymorphisms to evaluate the effects of QTL, and has more advantages than linkage analysis as it offers comparatively high-resolution power (which is based on the structure of LD) (Ibrahim et al., 2020) and provides the possibility to study various genomic regions simultaneously without construction of mapping populations (Saba Rahim et al., 2018). The size and diversity of the population for AM is critical to successful identification of key traits to previously known chromosomal regions with greater precision. The AM population must have sufficient variation for the traits of interest at both DNA sequence and phenotype levels. The greater is the size and extent of DNA sequence variation, the greater is the chance of discovering polymorphic markers (Liu et al., 2015). For instance, in one AM study, 104 peanut accessions were utilized to identify molecular markers associated with seed-related traits using 554 single locus simple sequence repeat (SSR) markers. Most of the accessions had weak or no relationship in the peanut panel, and large phenotypic variation was observed for four seed-related traits (seed length, seed weight, ratio of seed length to width, and hundred-seed weight) in the association panel (Zhao et al., 2017). AM detected a total of 30 significant SSR markers associated with four seed-related traits in different environments, which explained 11.22–32.30% of the phenotypic variation for each trait. The marker AHGA44686 was simultaneously and repeatedly associated with seed length and hundred-seed weight in multiple environments with large phenotypic variance (26.23∼32.30%), suggesting that AHGA44686 is a promising genetic marker which can enhance hundred-seed weight through seed length (Zhao et al., 2017). In soybean, Bao et al. (2015) used a set of 282 breeding lines (composed of ancestral lines, advanced breeding lines, released cultivars and landraces from the University of Minnesota Soybean Breeding Program) genotyped by using a genome-wide panel of 1536 SNP markers, to perform AM for four sudden death syndrome (SDS) (caused by *Fusarium virguliforme*) resistance traits (root lesion severity, foliar symptom severity, root retention, and dry matter reduction). AM approach identified significant peaks in genomic regions of known SDS resistance. Eight and two SNP markers in significant association with root retention and dry matter reduction were identified, respectively, exhibiting a total of five loci underlying SDS resistance, including three known SDS resistance QTL, viz., c*qSDS001* (on linkage group D2, chr 17), *c*qRfs4 (at position 80.28 cM on linkage group C2, chr 6), and SDS11-2, as well as two novel loci, *SDS14*-*1* (on chr 3) and *SDS14*-*2* (on chr 18). Interestingly, among the five loci identified, *cqSDS001* and *cqRfs4* had been previously identified and confirmed in multiple bi-parental populations, thereby strengthening the accuracy of the overall AM analysis (Bao et al., 2015). AM has also proved convenient in the identification of major-effect QTLs for grain yield under drought in rice (Swamy et al., 2017), heat tolerance in maize (Seetharam et al., 2021), and flowering time in rapeseed (Xu et al., 2015) among other important traits. Thus, aided by the recent developments in genome sequencing and computational tools, AM provides huge potential to enhance crop genetic improvement.

Meanwhile, multiparental, or next-generation mapping populations (NGMPs), possess greater utility as compared to biparental populations since they yield additional recombination break points and increase the allelic diversity and QTL detection power (Gangurde et al., 2020). Examples of NGMPs include nested-association mapping (NAM) (see Gangurde et al., 2020), Multi-parent Advanced Generation Inter-Cross (MAGIC) (Huang et al., 2015) and random-openparent association mapping (ROAM) (Xiao et al., 2016) (for extensive review, see Liang et al., 2021a; Sinha et al., 2021). These NGMPs can be effectively used to identify rare alleles in joint linkage association mapping studies to circumvent the limitations of natural mapping populations and GWAS. The recent genome sequenced and re-sequenced assemblies for various crop species are valuable resources for sequence based trait mapping and candidate gene discovery (Gangurde et al., 2020). Going forward, our focus is increasingly shifting from QTL identification to quantitative trait nucleotides (QTNs) and positional (or map-based) cloning. It is envisaged that in the near future fine mapping of QTLs and pinpointing of QTNs will become more efficient, consequently enhancing our capacity to perform precision breeding of crops that can withstand the emerging climatic shifts (Liang et al., 2021a; Varshney et al., 2021).

### Epigenomics as an Emerging Research Avenue for Abiotic and Biotic Stress Tolerance Breeding

Recently, epigenetics, which refers to the heritable and stable alterations in gene expression not attributable to DNA sequence changes or variation (Peschansky and Wahlestedt, 2014), has emerged as a potential research avenue for exploitation in our endeavor to develop climate smart crops (Crisp et al., 2021; Gogolev et al., 2021; Kakoulidou et al., 2021; Samantara et al., 2021). Such epigenetic modifications include DNA methylation, histone proteins/variants rearrangements, micro-RNA (mRNA) induced chromatin remodeling, histone acetylation, ATP-dependent nucleosome remodeling, among others (McCoy et al., 2021; Singh and Prasad, 2021). These epigenetic modifications are instituted to modulate spatio-temporal gene expressions in response to external stimuli or specific developmental requirements (Yuan et al., 2013; Singh and Prasad, 2021). More crucially, these epigenetic alterations involve the development of internal memory marks which assist plants to adapt to several abiotic and biotic stresses via physiological regulation directed by plants‘ epigenetic history (reviewed in Samantara et al., 2021; Sun et al., 2021). The molecular mechanisms underpinning plant environmental stress responses often rely on these epigenetic modifications (for extensive reviews, see Kim et al., 2010; Kim et al., 2015; Banerjee et al., 2017; Chang et al., 2020). A collection of examples of epigenetic studies for crop improvement are tabled in a more recent review by Kakoulidou et al., 2021. Therefore enhancing our understanding of the epigenetic regulation induced gene expressions related to abiotic and biotic stress responses will create more avenues for crop improvement for climate resilience via molecular breeding and/or biotechnological approaches (Chinnusamy et al., 2013; Singh and Prasad, 2021). Essentially, with the support of new genome analysis tools, epigenomics can be integrated with the investigation of non-coding RNA, cis-regulatory elements, and other non-genic variations controlling plant abiotic and biotic stress responses (Crisp et al., 2021; Zenda et al., 2021), to facilitate epigenetics-assisted breeding of crops (Gogolev et al., 2021).

## Omics Facilitated Crop Improvement for Abiotic and Biotic Stress Resistances

In this section, we shall briefly highlight, with several relevant examples, how the omics approaches and technologies have been successfully used in many studies focusing on abiotic and biotic stress responses in diverse crop species.

### Transcriptomics

Transcriptome profiling offers a global snapshot of the entire RNA molecules, including mRNA, tRNA, rRNA, sRNA, and other non-coding RNA within a cell, tissue, organ, or whole organism at any given time point, which is not possible to be investigated at the genomic level (Weckwerth et al., 2020; Chaturvedi et al., 2021). Understanding the transcriptome is crucial for deducing the genome‘s functional elements and revealing the molecular components of cells or tissues, understanding cells‘ responses to developmental and environmental stimuli triggered changes (Wang et al., 2009). Unlike the genome which is stable, the transcriptome is variable under different conditions (developmental stage, type of tissue, environmental stimuli, etc.), and is therefore a promising molecular level for exploring an organism’s stress responses (Kukurba and Montgomery, 2015; Escandón et al., 2021). Different technologies for deducing and quantifying the transcriptome have been established, including hybridization-or sequence-based methods (Wang et al., 2009). Such techniques are categorized as either targeted (microarray or reverse transcription-quantitative PCR (RT-qPCR) based) or untargeted (RNA-sequencing based) transcriptomic approaches (Escandón et al., 2021). Whereas hybridization-based methods usually encompass incubating fluorescently labeled cDNA with microarrays, sequence-based methods directly determine the cDNA sequence (for extensive review, see Wang et al., 2009).

These genome sequencing techniques have evolved over decades (see Section “High Quality Reference Genomes as Vital Resources for Accurate Annotation of Gene Structure, Content and Variation” above). Notably, the recent progress in high-throughput genome sequencing approaches and sequencing costs reduction has revolutionized the genomics research field. Particularly, this has brought about RNA-seq, a modern technique for both transcriptome mapping and quantification (Wang et al., 2009). Compared to other approaches, RNA-seq based method possesses several advantages of lower costs, a wider dynamic range, higher sensitivity, ability to provide whole-genome coverage, and applicability to non-model species (Kircher and Kelso, 2010; Chaturvedi et al., 2021), and has since provided unprecedented opportunities for conducting abiotic and biotic stress response studies in various crop species (Table 3). In particular, comparative transcriptomic approach has been widely applied in gene differential expression analysis in plants exposed to with- and without stress treatments in several crop species. For example, in a maize salinity stress response study, the tolerant genotype exhibited specific functional genes involved in salt tolerance, particularly CBL-interacting kinase (*Zm00001d044642*), salt stress induced protein (*Zm00001d023516*), thioredoxins (*Zm00001d018238*, *Zm00001d041804* and *Zm00001d018461*), defense genes such as leucine-rich repeat protein (*Zm00001d035756*) and pathogenesis-related protein (*Zm00001d018324*), and TF genes belonging to MYB (*Zm00001d053220*), WRKY (*Zm00001d005622*) and bZIP (*Zm00001d043992*) families, most of which were involved in the ABA signaling pathway (Zhang et al., 2021) and have been previously implicated in salt (Chen et al., 2013, 2014, Zhao C. et al., 2018) and drought (Zenda et al., 2019) stress tolerances. Besides, B73 maize plants grown under heat and control conditions revealed that several TF gene families including AP2-EREBP (*GRMZM2G010555*, *etc.*), b-ZIP (*GRMZM2G479760*, etc.), bHLH (*GRMZM2G001930*, *etc.*), and WRKY (*GRMZM2G324999*, *GRMZM2G071907*, *etc.*), and HSPs (*GRMZM2G069651*, *GRMZM2G366532*, *GRMZM2G149647*, etc.) were significantly enriched in the protein processing in endoplasmic reticulum (PPER) pathway, which played a key role in maize heat stress response (Qian et al., 2019). Moreover, Tifleaf 3 pearl millet genotype plants grown under heat and drought stress conditions showed that out of the nine ROS production related DEGs (two amine oxidases and seven polyamine oxidases), only two DEGs (*i2_LQ_LWC_c7872/f15p2/2851* and *i1_LQ_LWC_c34699/f1p0/1833*) were up-regulated in response to heat stress, suggesting the inhibition of ROS production after 48 hr of heat stress (Sun et al., 2020). Additionally, they identified five ROS scavenging enzymes, including SOD (*i0_LQ_LWC_c2218/f1p0/833*), CAT (*i2_HQ_LWC_c41068/f2p7/2070*), APX (*i1_LQ_LWC_c18498/f1p3/1627*, *i3_LQ_LWC_c37944/f1p0/3280*, etc.), and thirty HSPs (including *i2_HQ_LWC_c49563/f2p1/2825*, *i2_HQ_LWC_ c43630/f6p12/2432*, sHSP *i0_LQ_LWC_c967/f1p0/765*, etc.) that were up-regulated in response to heat stress (Sun et al., 2020). Under drought stress conditions, two *Asr* genes (*i1_LQ_LWC_c40079/f7p0/1159 and i0_HQ_LWC_c31/f2p0/781*) were up-regulated, suggesting the critical role of these LEA proteins in drought stress tolerance. Most of the genes were involved in photosynthesis, starch and sucrose metabolism, circadian rhythm, phenylpropanoid, and glycerophospholipid metabolic pathways (Sun et al., 2020).

**TABLE 3:** Selected examples of transcriptomic studies for abiotic and biotic stress tolerance in different crop species.

**Crop species**	**Genotypes used**	**Tissue analyzed**	**Sequencing strategy/platform used**	**Experiment type**	**Key findings**	**References**
**Abiotic stresses**						
**Drought stress**						
*Zea mays*	Susceptible RIL Mo17 and tolerant RIL Ye8112	Leaf	Illumina	Greenhouse	The tolerant genotype YE8112 drought-responsive genes were predominantly implicated in stress signal transduction, cellular redox homeostasis maintenance, carbohydrate synthesis and cell-wall remodeling, among others.	Zenda et al., 2019
*Oryza sativa*	Moderately tolerant line 4610 and susceptible Rondo	Leaf samples at grain-filling stage	Illumina	Field	The moderately tolerant genotype 4610 was less affected by drought stress due to its more rapid stress response and higher expression level of key drought-tolerant genes, LEA proteins, ROS scavengers, APXs and GSTs.	Liang et al., 2021b
*Triticum aestivum*	Drought-tolerant Colotana and sensitive Tincurrin	Root	Illumina	Lab	Several transcription factors, pyrroline-5-carboxylate reductase and late-embryogenesis-abundant (LEA) proteins were among the up-regulated genes in the tolerant cultivar Colotana responding to drought stress.	Derakhshani et al., 2020
*Glycine max*	Williams	Leaf	Illumina	Lab	The large number of DEGs and diverse pathways indicted that soybean employs complicated mechanisms to cope with drought	Xu C. et al., 2018
*Arachis hypogaea*	2 drought tolerant (C76-16 and 587 RILs) and 2 susceptible (Tifrunner and 506 RILs)	Leaf	Illumina Hiseq4000	Lab	Metabolic pathways involved in secondary metabolites biosynthesis, and starch and sucrose metabolism were highly enriched in tolerant cultivars in response to drought stress.	Wang X. et al., 2021
**Heat or heat and drought stress**						
*Oryza sativa*	Heat-tolerant Annapurna and sensitive IR64	Seedlings	Microarray-based	Growth chamber	The transcriptome analyses revealed a set of uniquely regulated genes and associated pathways in the tolerant genotype Annapurna, particularly associated with auxin and ABA as a part of heat stress response in rice.	Sharma E. et al., 2021
*Glycine max*	Heinong44	Leaf	Illumina	Lab	Many genes involved in the defense response, photosynthesis, and metabolic process were differentially expressed in response to drought and heat. Additionally, 1468 and 1220 up-regulated and 1146 and 686 down-regulated genes were confirmed as overlapping DEGs at 8 and 24 h after treatment	Wang L. et al., 2018
*Pennisetum glaucum*	Tifleaf 3	Seedling leaf and root	PacBio Sequel.	Growth chamber	Diverse genes were differentially expressed under heat and drought stresses, and comparing the DEGs under heat tolerance with the DEGs under drought stress, it was observed that even in the same pathway, pearl millet responds with a different protein	Sun et al., 2020
*Zea mays* (Sweet maize)	Heat-resistant Xiantian 5 and heat-sensitive Zhefengtian	Seedling leaf	Illumina HiSeq 2500	Growth chamber	Comparative transcriptomic profiling reveals transcriptional alterations in heat-resistant and heat-sensitive sweet maize varieties under heat stress, with the up-regulated DEGs mainly involved in secondary metabolite biosynthetic pathway	Shi et al., 2017
*Zea mays*	Inbred line B73 plants grown under heat and control conditions	Seedling leaf	Illumina	Growth chamber	Protein processing in endoplasmic reticulum pathway was observed to play a central role, and several TF families including MYB, AP2-EREBP, b-ZIP, bHLH, NAC and WRKY were associated with maize heat stress response.	Qian et al., 2019
**Salinity stress**						
*Gossypium hirsutum*	Salt-tolerant Zhong 07 and sensitive Zhong G5	Root	Microarray	Lab	Transcriptional regulation, signal transduction and secondary metabolism in two varieties showed significant differences, all of which might be related to mechanisms underlying salt stress tolerance in cotton.	Guo et al., 2015
*Triticum aestivum*	Xiaoyan 60 and Zhongmai 175	New leaf, old leaf, and root	Illumina	Lab	The most significantly enriched gene ontology (GO) terms and KEGG pathways were associated with polyunsaturated fatty acid (PUFA) metabolism in leaf tissues of Xiaoyan 60, whereas they were associated with photosynthesis and energy metabolism in Zhongmai 175.	Luo et al., 2019
*Cicer arietinum*	Tolerant (ICCV10, JG11) and susceptible (DCP93-2, Pusa256) genotypes	Root and shoot	Illumina Hiseq 2500	Hydroponic experiment	Under elevated salt stress conditions, tolerant genotypes activated a highly efficient response machinery involving enhanced signal transduction, transport and influx of K^+^ ions, and osmotic homeostasis	Kumar N. et al., 2021
*Zea mays*	Tolerant line L2010-3 and sensitive line BML1234	Seedling roots	Illumina	Growth chamber	The ABA signaling pathway likely coordinates the maize salt response process, and the tolerant genotype exhibited specific functional genes involved in salt tolerance, especially Aux/IAA, SAUR, and CBL-interacting kinases	Zhang et al., 2021
**Cold stress**						
*Zea mays*	21 DH genotypes from a DH population of 276 genotypes	Root	Illumina	Lab	The different genotypes showed highly variable transcriptome responses to cold stress	Frey et al., 2020
*Oryza sativa*	Cold-sensitive Ce 253 and tolerant Y12-4	Seed	Illumina	Greenhouse	There were more up-regulated DEGs in the cold-tolerant genotype than in the cold-sensitive genotype at the four stages under cold stress.	Pan et al., 2020
*Triticum aestivum*	Cold-tolerant Saratovskaya 29 and sensitive Yanetzkis Probat	Leaf	Illumina	Greenhouse	Groups of genes involved in response to cold and water deficiency stresses, including responses to each stress factor and both factors simultaneously were identified.	Konstantinov et al., 2021
**Metal toxicity stress**						
*Glycine max*	Aluminum (Al)-resistant (cv. PI416937) and Al-sensitive (cv. Huachun18)	Seedling roots	Micro-arrays	Pot experiment	The expression of a series of antioxidant enzymes related DEGs was induced in the Al-resistant cultivar than in Al-sensitive cultivar	Li et al., 2020
*Zea mays*	Zheng 58	Seedling roots	Illumina	Growth chamber	Increased auxin content and distribution in roots is required for cadmium (Cd) stress responses in maize	Yue et al., 2016
*Gossypium hirsutum*	Han242	Seedling root hairs, stalks, and leaf	Illumina	Greenhouse	*GhHMAD5*-silenced cotton plants showed more sensitivity to cadmium (Cd) stress, indicating that *GhHMAD5* is involved in Cd tolerance	Han et al., 2019
**Nutritional deficiency stress**						
*Zea mays*	Low P-tolerant line CCM454 and low P-sensitive line 31778	Seedling shoots and roots	Strand-specific RNA-seq, Illumina Hiseq 2500	Field	The tolerance to low P of CCM454 genotype was mainly attributed to the rapid responsiveness to P stress and efficient elimination of ROS	Du et al., 2016
*Triticum aestivum*	Nitrogen (N)-sensitive cultivar Shannong 29 grown under N deficient and N sufficient conditions	Seedling shoots and roots	Illumina HiSeqTM 2500	Hydroponic	48 candidate genes involved in improved photosynthesis and nitrogen metabolism were identified in wheat responses to nitrogen-deficiency	Liu X. et al., 2020
*Zea mays*	QPM inbred line SKV616 grown under iron (Fe) and zinc (Zn) deficiency	Seedling root and shoot	Micro-arrays	Hydroponic	Several DEGs, particularly those regulating Fe and Zn homeostasis were identified as candidate genes for enhancing Fe and Zn efficiency in maize	Mallikarjuna et al., 2020
**Biotic stresses**						
*Ipomoea batatas.* Lam	Zheshu 6025 genotype plants infected (VCSP) and non-infected (VFSP) with SPFMV, SPV2, and SPVG viruses	Seedlings	Illumina HiSeq 2500	Shed	Co-infection with SPFMV, SPV2, and SPVG viruses significantly reduced the expression of several genes involved in photosynthesis and photosynthesis-related pathways in VCSP	Shi J. et al., 2019
*Glycine max*	*Bacillus simplex* (strain Sneb545)-treated and non-treated Liao15 genotype plants under soybean cyst nematode (SCN)	Seedling roots	Illumina	Greenhouse	Key metabolic pathways including phenylpropanoid biosynthesis and cysteine and methionine metabolism were suggested to participate in the Sneb545-induced soybean response to SCN. Additionally, Sneb545-treated soybeans accumulated four nematicidal metabolites that inhibited SCN development	Kang et al., 2018
*Triticum aestivum*	Zhongmai175 genotype plants infected and non-infested with *S. graminum* aphids	Seedling leaf	Illumina HiSeq 4000	Climate chamber	Defense-related metabolic pathways and oxidative stress were rapidly induced in the tolerant genotype within hours after the initiation of aphid feeding.	Zhang Y. et al., 2020
*Cucumis melo* (melon)	Powdery mildew (*Podosphaera xanthii*) resistant MR-1 and susceptible Topmark cultivars	Leaf	Illumina	Controlled chamber	Several key genes and pathways involved in biotic resistance to *Podosphaera xanthii* powdery mildew were identified	Zhu et al., 2018
*Zea mays*	*Fusarium verticillioides* infested and non-infested plants of CML144 cultivar	Seedling leaf	Illumina	Culture room	Among the DEGs, *TPS1* and *cytochrome P450* genes were up-regulated, suggesting that kauralexins were involved in *Fusarium* ear rot defense response	Lambarey et al., 2020

In cotton, *GhHMAD5*-silenced cotton plants exhibited more sensitivity to cadmium (Cd) stress, demonstrating that *GhHMAD5* gene is involved in Cd tolerance (Han et al., 2019). In rice, the relatively tolerant genotype 4610 got less affected by drought stress than the susceptible genotype Rondo due to its more rapid stress response and higher expression of key drought-tolerance genes at the grain filling stage, including dehydrin rab (responsive to ABA) 16C (*Os11g0454000*) and Rab21 (*Os11g454300*), one bZIP TF (*Os01g0658900*), some known LEA proteins (*Os01g0705200*, *Os11g0454200*), ascorbate peroxidase (APX) (*Os04g0434800*), RIC2 family protein (*Os03g0286900*), drought and salt stress response 1 (*Os09g0109600*), and two HSP (*Os02g0232000*, *Os03g0277300*) genes (Liang et al., 2021b). In wheat, aphids (*Schizaphis graminum*) attack significantly increased the expression levels of several genes related to the salicyclic acid (SA) and jasmonic acid (JA) signaling pathways, including *lipoxygenase (LOX*, *TraesCS4B01G037700*, etc.), *FAD* (*TraesCS4A01G109300*, etc.), *phenylalanine ammonia-lyase* (*PAL*, *TraesCS2A01G196700*, etc.*)*, and *PR1* (TraesCS7D01G161200, TraesCS5A01G183300, etc.) genes (Zhang Y. et al., 2020). Additionally, several ROS scavenging enzymes such as POD (TraesCS2B01G125200, TraesCS2A01G107500, etc.), SOD (TraesCS2D01G123300) and CAT (TraesCS6A01G041700), as well as mitogen-activated protein kinases (Novel11623, TraesCS4D01G198600, etc.) and WRKY TF genes (*Novel00700*, *Novel01914*, etc.) were up-regulated in response to aphid attack (Zhang Y. et al., 2020). These results suggest that the SA, JA, protein phosphatases and MAPK-WRKY signaling pathways are the central metabolic pathways activated in response to aphid attack and can be targeted for aphid tolerance breeding. Thus, transcriptomic analysis has become central in abiotic and biotic stress tolerance studies (Li et al., 2019; Kaur et al., 2021; Table 3), and genes and metabolic pathways identified in these studies can be used as targets in marker assisted breeding programs.

With the large amount of data that has been generated and deposited into various public repositories, it is now possible to conduct meta-analysis of transcriptomic responses to abiotic and biotic stresses. It is now possible to acquire more reliable results by integrating information from multiple sources, and we can now study the expression and co-expression patterns of several genes under different abiotic stresses (Cohen and Leach, 2019; Tahmasebi et al., 2019). For instance, a meta-analysis of biotic and abiotic stress responses in tomato was performed by analyzing 391 microarray samples from 23 different experiments and 2,336 DEGs involved in multiple stresses were identified, including 1,862 DEGs responding to biotic and 835 DEGs responding to abiotic stresses, of which 4.2% of those DEGs belonged to various TF families (Ashrafi-Dehkordi et al., 2018). Among these TF genes, *Jasmonate Ethylene Response Factor 1* (*JERF1*), *MYB48*, *EIL2*, *EIL3*, protein LATE ELONGATED HYPOCOTYL (*LHY*), and *SlGRAS6* played critical roles in biotic and abiotic stress responses (Ashrafi-Dehkordi et al., 2018). Therefore, meta-analysis can be used for characterization and identification of candidate genes for both biotic and abiotic stress tolerance and the identified genes pinpointed as potential targets for the genetic engineering of improved stress tolerance crops.

Meanwhile, single cell transcriptomics (SCT) is slowly becoming the major omics approach for plant biology studies. Since its first assessment attempt in 2013, single cell transcriptome profiling has become an indispensable tool for decoding cell type, transcriptomic signatures, and performing single-cell transcriptomics of ncRNAs (Pratik, 2018). Although the greatest technical hurdles to adopting single-cell protocols to plants are related to dissociating cells from the appropriate tissues, obtaining sufficiently high numbers of cells for high-throughput analysis, the technical noise associated with single-cell assays, and the lack of true biological replicates (Efroni and Birnbaum, 2016), matching SCT analysis tools and algorithms are being developed to facilitate the use of SCT approach in molecular biology research (Gogolev et al., 2021). Recently, some researchers have used isolated protoplast or nuclei to successfully establish Arabidopsis roots and stomatal cells (Jean-Baptiste et al., 2019; Liu Z. et al., 2020), as well as maize anther cell transcriptomes (Nelms and Walbot, 2019; Xu et al., 2021) at the single-cell level (Thibivilliers and Libault, 2021). Further, single-cell ATAC-seq (Assay for Transposase Accessible Chromatin-sequencing) has been applied on nuclei isolated from Arabidopsis roots and different maize organs to divulge the differential chromatin accessibility between plant cell types (Thibivilliers and Libault, 2021). For instance, single cell RNA-seq has been applied to Arabidopsis root cells to capture gene expressions in 3121 root cells and hundreds of genes with cell-type–specific expressions were identified, revealing both known and novel genes that are expressed along the developmental trajectories of cell lineages (Jean-Baptiste et al., 2019). Additionally, single-nuclei RNA-seq has been integrated with ATAC-seq datasets to reveal how chromatin accessibility controls gene expression and the differential organization of the Arabidopsis genome between cell types (Farmer et al., 2021). As a result, these studies have shown the significant virtues of single-cell RNA-seq to detect rare cell types and resolve developmental trajectories in complex tissues, and have offered rare insights into the processes of cell differentiation, tissue-specific abiotic stress responses, cell-type-specific responses to genetic perturbations, and cell-cycle interactions (Denyer et al., 2019; Jean-Baptiste et al., 2019; Denyer and Timmermans, 2021). Thus, SCT approach is improving the spatiotemporal resolution of our analyses to the individual cell level, and is quickly expanding the portfolio of available tools and applications for plant molecular biology research (Rich-Griffin et al., 2020; Giacomello, 2021; Seyfferth et al., 2021). However, to harness the potential benefits of the SCT and to popularize its use in plant biology research, a lot of issues still need to be resolved, among which include the optimization of cell-isolation protocols, discerning the number of cells and sequencing reads required, and accommodating abiotic/biotic stress responses (Denyer and Timmermans, 2021).

### Proteomics

The proteomics domain involves the large-scale analysis of the proteome profile within an organism, tissue or cell, during normal organismal growth and development or in response to the fluctuations in environmental conditions. It aims to reveal the protein diversity, abundance, isoforms, localization, interactions with other proteins and post-translational modifications (PTMs) (Hashiguchi et al., 2010; Kosova et al., 2018; Labuschagne, 2018). It has been well acknowledged that the mRNA expressed at the transcriptional level is not directly linked with the plant phenotype; hence, it poorly correlates with the phenotype. However, the proteins are the direct effectors of the plant responses to developmental or environmental changes. Therefore, proteomics is a crucial link between transcriptomics and metabolomics (Tan et al., 2017; Labuschagne, 2018). Moreover, the proteome, unlike the genome which is static, is dynamic and the evaluation of proteins takes into account the effects of PTMs, thereby providing more information in understanding biological functions (Wu et al., 2016; Wu and Wang, 2016).

Several proteomics approaches have been deployed in molecular biology studies, and they are generally categorized into gel-based and gel-free-based techniques, coupled with mass spectrometry (MS) for protein identification, fractionation and analysis, as well as data processing techniques (reviewed in Mustafa and Komatsu, 2021; Sinha and Verma, 2021). On one hand, gel based proteomic approaches encompass initial protein separation by way of gel electrophoresis, followed by quantification, digestion and identification through MS. Examples of gel-based techniques include one or two dimensional polyacrylamide gel electrophoresis (1- or 2- DE) and differential in-gel electrophoresis (DIGE) (Tan et al., 2017; Labuschagne, 2018). On the other hand, gel-free technologies, which involve the digestion of intact proteins (via protease degradation) into peptides prior to liquid chromatographic (LC) separation and MS identification, include the isobaric tags for relative and absolute quantitation (iTRAQ), isotope-coded affinity tags (ICAT), and targeted mass tags (TMT), among others (extensively discussed in Chandramouli and Qian, 2009; Hu et al., 2015; Ghatak et al., 2017; Tan et al., 2017; Vo et al., 2021).

During the past two decades, the scientific community has witnessed tremendous advances in plant proteomics, largely characterized by the refinement of the conventional techniques and advent of modern, high throughput, and high-resolution approaches related to samples preparation and protein extraction, fractionation, quantification and analysis; proteomics data processing and analysis, among other areas (Ross et al., 2004; Matros et al., 2011; Agrawal et al., 2013; Tan et al., 2017). For instance, the proteomics field has seen a gradual shift from the general descriptive studies of plant protein abundances and covalent modifications to large scale analysis of protein-metabolite interactions (PMIs) and protein-protein interactions (PPIs) (Ramalingam et al., 2015; Scossa et al., 2021). These advances have been necessitated largely by the recent developments in LC-tandem MS systems, which have significantly improved their resolution and scanning rates. Particularly, the PMI field has been given special attention due to the role metabolites play, not only as metabolic intermediates, but also as co-factors or ligands with the capacity to alter protein confirmations and functions (Scossa et al., 2021). Detailed discussions on the advances made in plant proteomics can be accessed in numerous previous reviews (Agrawal et al., 2013; Ghatak et al., 2017; Kosova et al., 2018; Labuschagne, 2018; Raza et al., 2021a; Sinha and Verma, 2021).

Several next generation quantitative proteomic techniques have been widely employed in descriptive and comparative plant abiotic and biotic stress response studies (Ahmad et al., 2016; Mustafa and Komatsu, 2021). For instance, an iTRAQ-based comparative proteomics study to investigate the salinity-responsive proteins and related metabolic pathways in two contrasting rice genotypes at the maximum tillering stage identified 368 and 491 proteins that were up-regulated in the tolerant genotype LYP9 under moderate salinity and high salinity stress, respectively (Hussain et al., 2019). Among the highly expressed proteins were those involved in redox reactions, including peroxidases (gi| 125525683), glutathione -S- transferase (gi| 115459582) and SOD (gi| 125604340); salt stress-responsive proteins including malate dehydrogenase (gi| 115482534), methyltransferase (gi| 115477769), glucanase (gi| 13249140), pyruvate dehydrogenase (gi| 125564321), glutathione peroxidase (gi| 125540587), fructose-bisphosphate aldolase (gi| 218196772), and triosephosphate isomerase (gi| 125528336); photosynthesis related proteins including psbP-like protein 1 (gi| 38636895), thylakoid lumenal protein (gi| 115477166), ferredoxin-thioredoxin reductase (gi| 115447507), psbP domain-containing protein 6 (gi| 115440559), and photosystem II oxygen-evolving complex protein 2 (gi| 164375543); and carbohydrate metabolism related proteins such as xyloglucan endotransglycosylase/hydrolase protein (gi| 115475445), polygalacturonase (gi| 115479865) and β-glucosidase (gi| 115454825) (Hussain et al., 2019). In another comparative study, 2-DE proteomics analysis complemented with MALDI TOF mass spectrometry revealed 39 key proteins that mediate soybean response to heat stress, water stress and combined stresses, especially those involved in metabolism [alanine aminotransferase 2 (A8IKE5), glutamine synthetase (O82560), serine hydroxy methyl transferase 5 (C6ZJZ0), translation elongation factor (O23963), pyruvate dehydrogenase (E5RPJ6), etc.], response to heat [HSP70 (P26413), HSP22 (mitochondrial) (Q39818), HSP 17.6 kda class 1(P04795), 17.7 kda class 1 HSP (B4 × 941)], and photosynthesis [Rubisco activase (D4N5G3), oxygen-evolving enhancer protein 2 (I1JJ05), glyceraldehyde 3-phosphate dehydrogenase (Q2IOH4), chlorophyll A/B-binding protein (Q39831), etc.] showing significant cross-tolerance mechanisms in the tolerant genotype PI-471938 (Katam et al., 2020).

Further, a comparative iTRAQ proteomics analysis for wheat stripe rust (*Puccinia striiformis* f. sp. *tritici)* resistance in wheat cultivar Suwon11 revealed a set of ROS metabolism-related proteins, peptidyl–prolyl *cis–trans* isomerases (PPIases), RNA-binding proteins (RBPs), and chaperonins that were involved in the response to *Pst* infection (Yang Y. et al., 2016). Among the 42 ROS metabolism-related proteins (encompassing GPXs, CATs, and peroxiredoxins), 11 peroxidases were strongly induced at both 24 and 48 hpi. Twelve PPIases (including AEGTA05000, AEGTA08970, AEGTA26095, AEGTA06390, etc.) were strongly up-regulated at 24 hpi. Moreover, thirteen RBPs, including one alternative splicing regulator (AEGTA28251), one arginine/serine-rich splicing factor (TRAES3BF080700020CFD_c1) and two predicted glycine-rich RBPs (AEGTA28395 and TRAES3BF152900030CFD_c1) were significantly altered (by exhibiting up-regulation) during the incompatible interaction, particularly at 24 hpi. Further, six chaperonins were also up-regulated at 24 hpi (Yang Y. et al., 2016). Besides, a comparative label-free quantitative proteomic analysis of three sorghum genotypes with variable resistance to spotted stem borer (*Chilo partellus*) insect pest identified putative leaf *C. partellus* responsive proteins. Among a total of 967 *C. partellus*-responsive proteins, those involved in stress and defense, photosynthesis, small molecule biosynthesis, amino acid metabolism, catalytic and translation regulation activities were significantly up-regulated in resistant sorghum genotypes upon pest infestation (Tamhane et al., 2021). Especially, known defense proteins such as pathogenesis related protein 5 (PR-5), thaumatin like pathogenesis related protein 1, chitin-binding type-1 domaincontaining protein, osmotin, calmodulin, peroxidases, glutathione S-transferase, expansin-like EG45 domaincontaining protein, non-specific lipid transfer protein, abscisic acid stress ripening 3, and alpha-amylase/trypsin inhibitor were amongst the candidate proteins identified (Tamhane et al., 2021), strengthening their role in plant defense against insect pest and pathogen attack (War et al., 2012; Zhang et al., 2018). Other proteomics studies aimed at identifying key proteins associated with responses to several abiotic and biotic stresses are available (Hu et al., 2015; Luo et al., 2018; Table 4). Overall, the information generated from these proteomic studies can be an invaluable resource for crop breeding programs, as it facilitates for potential markers identification, candidate proteins isolation and incorporation into breeding pipelines via proteomics-driven-marker assisted selection and protein-marker-centered gene pyramiding (Agrawal et al., 2012; Labuschagne, 2018).

**TABLE 4:** Examples of proteomic studies for abiotic and biotic stress tolerance in different crop species.

**Crop species^1^**	**Genotypes used^2^**	**Tissue analyzed**	**Strategy^3^**	**Experiment type**	**Major findings^4^**	**References**
**Abiotic stresses**						
**Drought or water deficit stress**						
*Zea mays*	Susceptible RIL Mo17 and tolerant RIL Ye8112	Seedling leaf	iTRAQ	Greenhouse	Better drought tolerance of the resistant genotype YE8112 was attributed to its activation of photosynthesis related proteins, and increased cellular detoxification capacity.	Zenda et al., 2018
*Phaseolus vulgaris*	Drought-tolerant Tiber and drought-sensitive Starozagorski èern	Leaf	2D-DIGE	Pot, controlled environ.	Energy metabolism, photosynthesis, ATP interconversions, protein synthesis and proteolysis, stress and defense related DAPs responded to drought stress especially in the tolerant genotype	Zadražnik et al., 2013
*Zea mays*	Drought-tolerant Chang 7-2 and sensitive TS141	Seedling root	iTRAQ	Greenhouse	The higher drought tolerance of Chang 7-2 root system was attributed to a stronger water retention capacity, the synergistic effect of antioxidant enzymes, and the osmotic stabilization of plasma membrane proteins.	Zeng et al., 2019
*Vigna unguiculata*	Water deficit stress-tolerant Pingo de Ouro 1,2 and sensitive Santo Inácio	Leaf	2D-PAGE	Greenhouse	108 DAPs associated with drought response in both genotypes were identified, with drought stress-response peptides, including glutamine synthetase, CPN60-2 chaperonin, malate dehydrogenase and HSPs being expressed differentially in both genotypes.	Lima et al., 2019
*Sorghum bicolor*	Drought-sensitive ICSB338 and drought-tolerant SA1441	Seedling root	iTRAQ	Pot, growth chamber	Root proteome analysis revealed common and unique proteins differentially accumulated in the two sorghum genotypes in response to water limitation.	Goche et al., 2020
**Heat, or high temperature, or combined heat and drought stress(es)**						
*Oryza sativa*	Heat-tolerant N22 and sensitive Mianhui101 cultivars	Anthers	iTRAQ	Pot in-field	Heat stress induced increased expression of sHSP, β-expansins and lipid transfer proteins in the resistant genotype N22, which might contribute to its ability to tolerate heat stress.	Mu et al., 2017
*Glycine max*	Heat-tolerant PI-471938 and heat-sensitive R95-1705	leaf	2-DE + MALDI TOFMS	Growth chamber	DAPs were elevated in high abundance to combined heat and water stresses in the tolerant genotype PI-471938 demonstrating enhanced promotive interactions associated with metabolism and photosynthesis which led to continued resistance to both types of stresses.	Katam et al., 2020
*Triticum aestivum*	Chinese Spring cultivar	Leaf	iTRAQ	Greenhouse	258 heat-responsive proteins (HRPs) involved in several biological pathways such as chlorophyll synthesis, carbon fixation, protein turnover, and redox regulation were identified.	Lu et al., 2017
*Glycine max*	Surge and Davison under drought and heat	Leaf	2D-DIGE	Growth chamber	Higher abundance of heat stress-induced EF-Tu protein, photosynthesis-related proteins, and HSPs was observed in the genotype Surge, probably activating soybean heat tolerance.	Das et al., 2016
*Capsicum annuum*	Heat-tolerant 17CL30 and sensitive 05S180	Seedling leaf	iTRAQ	Growth chamber	1,591 DAPs were identified as heat-responsive proteins, and were involved in photosynthesis, endoplasmic reticulum, porphyrin and chlorophyll metabolism pathways, among others.	Wang J. et al., 2021
**Salinity stress**						
*Glycine max*	Salt-sensitive Jackson and salt-tolerant Lee 68	Seedling leaf	2-DE coupled with MS/MS	Hydroponic	Tolerant genotype Lee 68 exhibited higher ROS scavenging ability, abundant energy supply and ethylene production, and stronger photosynthesis capacity than sensitive genotype Jackson under salt stress	Ma et al., 2012
*Zea mays*	Salt-tolerant 8723 and salt-sensitive P138 RILs	Root	iTRAQ	Greenhouse	Compared to the P138, the root responses of the tolerant genotype 8723 could maintain stronger water retention capacity, metabolism and energy supply capacity, osmotic regulation ability, and ammonia detoxification ability.	Chen et al., 2019
*Cicer arietinum*	Salt-tolerant Flip 97-43c and salt-sensitive Flip 97-196c	Seedling leaf	2-DE along with LC-MS/MS	Greenhouse	The differential salinity response in the tolerant and sensitive genotypes could be related to the reprogramming of several DAP expression patterns that induce changes in energy metabolism, including photosynthesis, stress-responsive proteins, protein processes, and signaling.	Arefian et al., 2019
*Oryza sativa*	Sensitive Nipponbare and tolerant LYP9	Seedling root and leaf	iTRAQ	Greenhouse	The DAPs up-regulated in response to salt stress were mainly involved in oxidation-reduction, photosynthesis, and carbohydrate metabolism processes.	Hussain et al., 2019
**Cold, flooding or water-logging (hypoxic) stresses**						
*Glycine max*	Cold-tolerant Guliqing and cold-sensitive Nannong 513	Seedling leaf	2-DE, coupled with MALDI-TOF/TOF MS	Pot in field	57 protein spots were significantly changed in abundance in response to cold stress, and were involved in several metabolic pathways such as photosynthesis, protein folding and assembly, cell rescue and defense, CHO metabolism, lipid metabolism, and energy metabolism, among others. Greater cold tolerance of Guliqing was attributed to its higher protein, lipid and polyamine biosynthesis, and higher photosynthetic rates than the sensitive genotype.	Tian et al., 2015
*Brassica rapa*.	Cold-tolerant Longyou 7 and cold-sensitive Tianyou 4	Leaf	iTRAQ	Pot, in artificial climate	Decreased abundance of most DAPs involved in ribosomes, carbon metabolism, photosynthesis, and energy metabolism was greater in cold-stressed Longyou 7 than in cold-stressed Tianyou 4. Thus, decreased energy metabolism, together with decreased photosynthesis, enabled winter turnip rape to balance synthesis and consumption of sugar, and better acclimate to cold stress.	Xu Y. et al., 2018
*Hordeum vulgare*	Water-logging sensitive TF57 and tolerant TF58.	Seedling leaf, and roots – adventitious, nodal & seminal	TMS	Pot, greenhouse	Among the key DAPs responding to hypoxic stress, photosynthesis-, metabolism- and energy-related proteins were diferentially expressed in the leaves, with oxygen-evolving enhancer protein 1, ATP synthase subunit and HSP 70 being up-regulated in tolerant genotype TF58.	Luan et al., 2018
**Metal toxicity stress**						
*Hordeum vulgare*	Tibetan wild annual Al-tolerant XZ16 and Al-sensitive XZ61, and Al-resistant cv. Dayton	Seedling root	2-DE analysis	Hydroponic	Four proteins (SAMS3, ATP synthase beta subunit, TPI, and Bp2A protein), were exclusively expressed in XZ16, but not in Dayton and XZ61 under Al stress, indicating their crucial role in development of Al stress tolerance in XZ16.	Dai et al., 2013
*Arachis hypogaea*	Low cadmium (Cd) cultivar Fenghua 1 and Cd cultivar Silihong	Seedling root	iTRAQ	Hydroponic, growth chamber	Several DAPs that may be involved in vacuolar sequestration of Cd and its efflux from symplast to apoplast, as well as cell wall modification, were up-regulated in Silihong in response to Cd exposure, thereby increasing Silihong‘s Cd uptake and sequestration capacity.	Yu R. et al., 2019
*Sorghum bicolor*	Inbred line BTx623 under cadmium (Cd) and non-Cd conditions	Seedling leaf and root	2-DE	Growth chamber	Out of the 33 differentially expressed protein spots (DEPS) analyzed, 15 DEPS showed increased, whilst 18 DEPS showed decreased expression in response to Cd exposure. Major proteomic alterations were observed in proteins involved in CHO metabolism, transcriptional regulation, translation and stress responses.	Roy et al., 2016
**Nutritional deficiency stress**						
*Glycine max*	HN66 under low P conditions	Shoots, roots, nodules	MALDI TOF/TOF MS analysis	Hydroponic	Several DAPs were significantly altered in response to Pi starvation, including malate dehydrogenase, ascorbate peroxidase and heat-shock proteins. Additionally, nodules response to Pi starvation was suggested to differ from those of roots response.	Chen et al., 2011
*Zea mays*	Inbred line Qi319 under low P conditions	Seedling shoots and roots	2-DE	Greenhouse	Maize developed different ROS scavenging strategies to cope with low P stress, including up-regulating its antioxidant content and antioxidase activity.	Zhang et al., 2014
*Triticum aestivum*	Aluminum (Al)-tolerant Atlas 66 and Al-sensitive Scout 66 cultivars under N deficiency	Seedling root and shoot	NanoLC-ESI-MS/MS	Hydroponic	Sensitive line Scout 66 had greater proteomic changes than tolerant line Atlas 66, with the majority of DAPs being enriched in cellular N compound metabolic process and photosynthesis processes.	Karim et al., 2020
**Biotic stresses**						
*Triticum aestivum* ^1^	Suwon11	Leaf	iTRAQ	Controlled chamber	Peptidyl–prolyl *cis–trans* isomerases (PPIases), RNA-binding proteins (RBPs), and chaperonins were the key DAPs involved in regulating wheat immune response to *Pst* infection.	Yang Y. et al., 2016
*Gossypium hirsutum*	*Rhizoctonia solani* tolerant cultivar CR135	Seedling root	iTRAQ	Controlled chamber	174 DAPs were identified to respond to *R. solani* infection, most of which these DAPs were involved in ROS homeostasis, epigenetic regulation and phenylpropanoid biosynthesis pathways, which were tightly linked with the innate immune responses against *R. solani* infection in cotton.	Zhang M. et al., 2017
*Zea mays*	Inbred line B73 seedlings under RBSDV infection	Shoots	LC-MS/MS coupled with TMT	Greenhouse	Key maize DAPs responding to RBSDV infection, including two sulfur metabolism-related proteins, were enriched in various metabolic pathways such as cyanoamino acid metabolism, protein processing in endoplasmic reticulum, and ribosome-related pathways.	Yue et al., 2018
*Oryza sativa*	RBD resistant GY8 and susceptible LTH	Seedling leaf	iTRAQ	Paddy field	The pathogen-associated molecular pattern (PAMP)-triggered immunity defense system could be activated at the transcriptome level but was inhibited at the protein level in susceptible rice variety after inoculation	Ma et al., 2020
*Sorghum bicolor*	Spotted stem borer- (*Chilo partellus)* resistant ICSV700 and IS2205; and susceptible Swarna	leaf	LC-MS/MS	Field	Several DAPs responding to *C. partellus* infestation were identified in resistant genotypes, including those involved in stress and defense, small molecule biosynthesis, amino acid metabolism, catalytic and translation regulation activities.	Tamhane et al., 2021

***Species:** ^1^Wheat cultivar Suwon11 plants inoculated or uninoculated with the avirulent *Puccinia striiformis* f. sp. *tritici* (*Pst*), race CYR23.*

*^2^**Genotypes used:** RIL, recombinant inbred line; RDB, Rice blast disease caused by *Magnaporthe oryzae (M*. *oryzae)*; RBSDV, Rice black streaked dwarf virus.*

*^3^**Strategy:** iTRAQ, isobaric tags for relative and absolute quantification; 2-DE, two-dimension al electrophoresis; 2D-DIGE, two-dimensional difference in gel electrophoresis; 2D-PAGE, two-dimensional gel electrophoresis; MS/MS, tandem mass spectrometry; MALDI TOF/TOF MS, DNA Matrix-assisted laser desorption/ionization time-of-flight mass spectrometry; TMT, Tandem Mass Tag labeling; LC-MS/MS, liquid chromatography–mass spectrometry/mass spectrometry; NanoLC-ESI-MS/MS, nano liquid chromatography – electrospray ionization – tandem mass spectrometry.*

*^4^DAP, differentially abundant/accumulated protein.*

Meanwhile, protein PTMs such as phosphorylation, nitrosylation and ubiquitination are central in the modulation of several cellular functions in plants, including metabolism, signaling transduction, gene expression, protein stability and interactions, and enzyme kinetics, as well as plant-environmental interactions (Kaufmann et al., 2011; Hashiguchi and Komatsu, 2017; Tan et al., 2017). Therefore, systematic investigations of these PTMs is critical for gaining insights into several regulatory mechanisms underpinning biological processes, including plant stress responses (Tan et al., 2017). Fortunately, the study of protein PTMs is increasingly gaining attention in plant science, particularly on their role in abiotic stresses (Wu et al., 2016; Haak et al., 2017; Stone, 2019; Martí et al., 2020) and plant immunity (De Vega et al., 2018; Zhang and Zeng, 2020). This is being driven by MS-based identification and analytical approaches in targeted proteomics (extensively reviewed in Arsova et al., 2018), as well as new innovations to study complex PTMs and integrate them with other domains such as epigenetics (Wu et al., 2016). For instance, MS-based analysis of chromatin has emerged as an indispensible tool for the identification of proteins linked to gene regulation, as it facilitates studying of protein functions and protein complex formation in their *in vivo* chromatin-bound context (van Mierlo and Vermeulen, 2021). Going forward, our ability to identify and quantify PTMs, supported by robust, efficient and high-throughput analytical and computational tools, will facilitate for large-scale comprehensive protein functional characterization that will enhance our knowledge of the crop stress acclimation and tolerance acquisition (Wu et al., 2016; Arsova et al., 2018).

### Metabolomics

In response to various environmental and pathogenic stresses, plants institute sophisticated physiological, biochemical and molecular mechanisms, including biosynthesis of a diverse range of metabolites, antioxidant enzymes activation, ions uptake and transport, osmoprotectants (especially proline) accumulation, and phytohormones release, among others (Pandey et al., 2015; Singhal R.K. et al., 2021). Metabolites encompass hundreds or thousands of primary or secondary compounds such as organic acids, sugar alcohols, hormones, allelochemicals, ketones, amino acids, steroids, etc. (Razzaq et al., 2019; Singhal R.K. et al., 2021). More crucially, plants have been observed to undergo metabolic adjustments in order to acclimate to predominant stress conditions by synthesizing anti-stress components including antioxidants, compatible solutes and stress-responsive proteins (Ramalingam et al., 2015). Therefore, metabolomics is aimed at qualitatively and quantitatively detecting, quantifying and analyzing all low molecular weight metabolites (called metabolome) within a cell, tissue, or an organism synthesized via cellular metabolism at a specific developmental stage, and/or in response to certain environmental stimuli (Fiehn, 2002; Arbona et al., 2013).

Owing to their close link to the phenotypic expression more than the mRNA transcripts and proteins, metabolites more precisely reflect the connection between gene expressions, protein interactions and diverse regulatory processes, as well as offering a direct functional readout of the physiological state of the cell (Arbona et al., 2013; Ramalingam et al., 2015; Pinu et al., 2019). Therefore, metabolomics, integrated with mass spectrometric and bioinformatics analyses, is an indispensable tool to study plant molecular responses to abiotic and biotic stresses, since alterations in the flux of both primary and secondary metabolites can be observed and analyzed against several stress conditions (Singh N. et al., 2021). Thus, in a bottom-up approach of omics integration, metabolomics data can be used to target subsequent up-stream proteomics or transcriptomics analyses to uncover mechanistic genes or proteins driving the processes of plant responses to stresses (Saito and Matsuda, 2010; Pinu et al., 2019). In other words, metabolomics is a more appropriate foundation for developing plant phenotype biomarkers and cross-omics biomarkers since it integrates genetic and non-genetic factors (Jendoubi, 2021).

Major plant metabolomics methods comprise metabolite profiling (focusing on metabolites with similar and specific chemical properties, and requires separation techniques), metabolic fingerprinting (without the need for separation technique, and uses different kinds of analyzers to compare sets of spectra and hence the samples from which the spectra were derived), and targeted analysis (identification and quantitative analysis of targeted metabolic compounds) (Krishnan et al., 2005; Arbona et al., 2013; Ramalingam et al., 2015). These approaches can be applied individually or in integration depending on the objective of the study (Ramalingam et al., 2015).

Most notably, the post-genomics period has seen massive improvements in the traditional (separation and MS based) methods to cutting-edge technologies that are facilitating for cost-efficient and high-throughput ways for molecular detection, quantification and analysis of a diverse range of metabolites (Kumar et al., 2017; Scossa et al., 2021). It is not surprising that the metabolomics domain is fastly receiving attention in both basic and applied plant research. More specifically, the advent of “hyphenated” separation methods and several detection systems has facilitated for systematic detection, quantification and analysis of a vast array of plant metabolites (Fraire-Velázquez and Balderas-Hernández, 2013). Liquid chromatography (LC), gas chromatography (GC) and capillary electrophoresis (CE) comprise the separation methods, whereas different types of MS, including MS, LC-MS, flow injection analysis coupled to MS (FIA/MS), ultraviolet light spectroscopy (UV/VIS), nuclear magnetic resonance (NMR), and high resolution mass spectrometry (HRMS) technologies are used for detection (Arbona et al., 2013; Fraire-Velázquez and Balderas-Hernández, 2013; Li et al., 2019). Direct infusion mass spectrometry (DIMS) and Fourier transform ion cyclotron resonance mass spectrometry (FT-ICR-MS) are specialized techniques normally used in direct infusion mode for metabolomics analyses since their high mass accuracy permits for separation to be achieved entirely based on this parameter (Fraire-Velázquez and Balderas-Hernández, 2013; Villate et al., 2021). Applicability and limitations of these metabolomics methods and techniques have been extensively discussed in previous articles (Allwood et al., 2011; Razzaq et al., 2019; Hamany Djande et al., 2020; Kaur et al., 2021).

Crucially, over the past decade, metabolomics approaches have facilitated for data mining and interpretation for structural elucidation of complex biological networks underpinning plants‘ responses to abiotic and biotic stresses (Saito and Matsuda, 2010; Resham et al., 2014; Barupal et al., 2018; Sharma V. et al., 2021). For instance, a comparative metabolic investigation of drought stress tolerance in contrasting groundnut genotypes using GC-MS, HPLC and UPLC-MS/MS analyses identified 46 key drought responsive metabolites (including pentitol, phytol, xylonic acid, d-xylopyranose, stearic acid, and d-ribose, agmatine, cadaverine, etc.). Among these, agmatine and cadaverine were accumulated in both roots and leaves, and were suggested as potential polyamines for drought tolerance (Gundaraniya et al., 2020). Additionally, seven metabolic pathways (including galactose metabolism, starch and sucrose metabolism, pentose and glucuronate interconversion, etc.) were revealed as critical in groundnut response to drought stress (Gundaraniya et al., 2020). These findings can augment transcriptomic and proteomic inquiries aimed at improving drought tolerance in groundnut. Besides, metabolomic profiling of soybean leaf tissues by GC-MS and LC-MS analyses revealed the role of phytochemical metabolism, as well as sugar and nitrogen metabolism in conferring tolerance to combined drought and heat stress conditions (Das et al., 2017). Integrated metabolomic, transcriptomic and gene regulatory network analyses of common rust (*Puccinia sorghi*) resistance in maize identified a number of *Rp1-D*-mediated defense response metabolites (including chlorogenic acid, caffeic acid, ferulic acid, flavonoids, terpenoids, kauralexins and zealexins) and genes involved in SA biosynthesis (especially, calmodulin-binding protein 60G and systemic acquired resistance deficient 1, *SARD 1;* and several TFs such as WRKY53, BZIP84, NKD1, BHLH124 and MYB100) as potentially critical regulators of *P. sorghi* resistance in maize (Kim S.B. et al., 2021). Additionally, they revealed a number of secondary metabolite biosynthesis (especially “phenylpropanoid and phenolics” and “terpenoid biosynthesis”) pathways as key in modulating common rust defense response in maize (Kim S.B. et al., 2021). Further, metabolic profiling of root lesion nematode (*Pratylenchus thornei*) resistant and susceptible wheat genotypes using UHPLC-QTOF analysis revealed that metabolites belonging to the fatty acids, flavonoid, glycerolipid, alkaloids, and steroid glycoside classes were constitutively expressed in the resistant wheat genotype (QT16258) roots (Rahaman et al., 2021), suggesting that the induction of these compounds in roots is a part of the inducible chemical arsenal that wheat employs to counteract root lesion nematode infection. Besides these few examples highlighted here, several other metabolic studies for crop improvement are listed (Table 5) and reviewed (Kumar et al., 2017; Kaur et al., 2021; Singhal R.K. et al., 2021; Vo et al., 2021). Taken together, metabolic profiles identified from these comparative studies can fortify transcriptomics and proteomics findings or can be utilized as signatures for evaluating the genetic diversity among different cultivars or species of the same genotype at different crop growth phases and environments and could guide tailoring of genotypes for desired or targeted performance under specific growth conditions, i.e., designing and creating crop varieties best suited to specific agricultural environments (Fraire-Velázquez and Balderas-Hernández, 2013).

**TABLE 5:** Selected examples of metabolomics studies to help understand abiotic and biotic stress tolerance mechanisms in different crop species.

**Crop species**	**Genotypes used**	**Stress Condition^1^**	**Tissue/s analyzed**	**Strategies/platforms used to analyze samples^2^**	**Data analysis methods used^3^**	**Key findings**	**References**
**Abiotic stresses**							
*Arachis hypogaea*	Tolerant TAG24 and sensitive JL24	Drought	Leaf and root	GC–MS, HPLC, UPLC–MS/MS	PCA, PLS-DA, HMp, CA	46 metabolites including pentitol, phytol, xylonic acid, d-xylopyranose, etc. were identified as key drought-responsive metabolites. Seven metabolic pathways, including galactose metabolism, starch and sucrose metabolism, fructose and mannose metabolism, propanoate metabolism, etc. were significantly affected by drought.	Gundaraniya et al., 2020
*Hordeum vulgare*	Tolerant Clipper cultivar and sensitive Sahara, a North African landrace	Salinity	Root	GC-MS	HMp	76 known metabolites, including 29 amino acids and amines, 20 organic acids and fatty acids, and 19 sugars and sugar phosphates were identified as key salt-responsive metabolites. Conclusively, the maintenance of cell division in the tolerant genotype responding to short-term salt stress was associated with the synthesis and increased accumulation of amino acids (proline), sugars (maltose, sucrose, xylose), and organic acids, suggesting a potential role of these metabolic pathways in barley salt tolerance	Shelden et al., 2016
*Glycine max*	Williams-82 cultivar	Heat and drought	Leaf	GC-MS, LC-MS	PCA, HMp, HCA	Conclusively, metabolomic profiling demonstrated that in soybeans, keeping up with sugar and nitrogen metabolism is of prime significance, along with phytochemical metabolism under drought and heat stress conditions	Das et al., 2017
*Cicer arietinum*	Sensitive Punjab Noor-2009 and tolerant 93127	Drought	Leaf	UPLC-HRMS	SAM, PLS-DA	Twenty known metabolites were identified as key drought-responsive metabolites, with proline, _L_ -arginine, _L_-histidine, _L_-isoleucine, and tryptophan exhibiting increased accumulation in the tolerant genotype after drought induction. Additionally, aminoacyl-tRNA and plant secondary metabolite biosynthesis and amino acid metabolism pathways were involved in producing genetic variation under drought conditions.	Khan et al., 2019
*Oryza sativa*	02428 (*japonica*) and YZX (*indica*)	Low temperature (cold)	Germinating seeds	LC–MS/MS, LC-ESI-MS/MS	PCA, PLS-DA	35 different metabolites that responded to cold stress were identified, among which 7 metabolites were defined as key metabolites, and were involved in the biosynthesis of amino acids and phenylpropanoids, and glutathione and inositol phosphate metabolism.	Yang et al., 2019
*Triticum aestivum*	Sensitive Frument and tolerant Jackson cultivars	Submergence	Shoots	GC QTOF MS, LC-MS, LC QTOF MS	PCA, ANOVA	Elevated levels of MDA suggested that the sensitive genotype Frument experienced higher levels of ROS-inflicted membrane damage at the end of the submergence period, whereas greater accumulation of proline in tolerant genotype Jackson may have contributed to the suppression of lipid peroxidation during submergence.	Herzog et al., 2018
*Sorghum bicolor*	Tolerant Samsorg 17 and sensitive Samsorg 40	Drought	Leaf	FT-IRS, non-targeted GC-MS	PCA, PC-DFA	A total of 188 compounds, with 142 known metabolites and 46 unknown small molecules, were detected in the two sorghum varieties. Conclusively, the two genotypes adopted distinct approaches in response to drought. Whilst Samsorg 17 accumulated sugars and sugar alcohols, Samsorg 40 exhibited increased accumulation in amino acids under drought stress conditions.	Ogbaga et al., 2016
**Biotic stresses**							
*Oryza sativa*	Resistant 32R and susceptible 29S lines	*Rhizoctonia solani* infection	Leaf	CE/TOF-MS in negative ion mode	MPP software	*R. solani* infection induced significant increases in adenosine diphosphate, glyceric acid, mucic acid and jasmonic acid in the resistant genotype 32R. Conclusively, *R. solani* infection effects in 32R were associated with the induction of plant metabolic processes such as respiration, photorespiration, pectin synthesis, and lignin accumulation.	Suharti et al., 2016
*Gossypium hirsutum*	Susceptible CIM-573 and resistant NIA-Sadori cultivars	*Aspergillus tubingensis* infection	Leaf	UPLC-MS	PCA, OPLS-DA, PLS-DA	Metabolite profiling revealed abundant accumulation of key metabolites including flavonoids, phenylpropanoids, terpenoids, fatty acids and carbohydrates, in response to cotton leaf spot. Among the 241 resistance related metabolites, 18 were identified as resistance related constitutive (RRC) and 223 as resistance related induced (RRI) metabolites. Several identified RRI metabolites were the precursors for many secondary metabolic pathways, and secondary metabolism, primary metabolism and energy metabolism were more active in resistant cultivar than in the sensitive cultivar.	Khizar et al., 2020
*Solanum lycopersicum*	Rutgers cultivar	CEVd and *Pseudomonas syringae* infection	Leaf	NMRS	PCA, PLS-DA	A large number of primary and secondary metabolites were identified in response to viroid and bacterial infection. While glycosylated gentisic acid was the most important induced metabolite in the viroid (CEVd) infection, phenylpropanoids and a flavonoid (rutin) were found to be associated with bacterial (*Pseudomonas syringae*) infection.	López-Gresa et al., 2010

*^1^Stress condition: CEVd, *Citrus exocortis* viroid.*

*^2^Strategies: GC-MS, gas chromatography–mass spectrometrchy; HPLC, high-performance liquid chromatography; UPLC-MS/MS, ultrahigh-performance liquid chromatography–tandem mass spectrometry; FT-IRS, Fourier transform infrared spectroscopy; CE/TOF-MS, capillary electrophoresis/tandem time-of-flight coupled with mass spectrometry; GC QTOF MS, gas chromatography quadrupole time-of-flight mass spectrometry; LC-ESI-MS/MS, liquid chromatography-electrospray ionization- -tandem mass spectroscopy; NMR, nuclear magnetic resonance spectroscopy.*

*^3^Data analysis methods: PCA, principal component analysis; PLS-DA, partial least-squares discriminant analysis; HMp, heat map; CA, cluster analysis; HCA, hierarchical clustering analysis; SAM, significant analysis of metabolites; PC-DFA, principal component discriminant function analysis; OPLS-D, orthogonal partial least squares discriminant analysis; MPP software, Mass Profiler Professional software (Agilent Technologies, Santa Clara, CA, United States).*

Large-scale metabolite profiling is offering convenience in accessing the global metabolites data sets and their corresponding metabolic pathways in an unparalleled way (Kumar et al., 2017). Thus, plant metabolomics has provided gateways in the discovery of new metabolic pathways and its integration with other omics has improved existing genome annotations. Moreover, metabolic-based quantitative trait loci (mbQTL) mapping is fastly proving to be an effective approach for identifying stress-responsive trait pathways (reviewed in Sharma V. et al., 2021). Complementary to genetic QTLs, proteomic QTLs and epigenetic QTLs, mbQTLs are also employed for quantitative traits mapping and identification of genetic variations at the metabolic level. Consequently, GWASs based on mbQTLs and metabolomics GWAS (mbGWAS) have become key in detecting genetic variations associated with metabolic traits in plants, thereby facilitating metabolomics-assisted breeding of crops (reviewed in Razzaq et al., 2019; Kumar R. et al., 2021). For instance, a metabolic profiling of barley flag leaves under drought stress conditions identified 57 mbQTLs for metabolites linked to primary carbon and nitrogen metabolism, as well as antioxidant metabolism pathways. Interestingly, mbQTLs for flag leaf γ-tocopherol, glutathione and succinate content were observed (by association mapping) to co-localize with the genes encoding enzymes of the pathways synthesizing these antioxidant metabolites (Templer et al., 2017).

Looking ahead, embracing the current trends in new technologies and approaches in crop biotechnology, the metabolite investigation of mutants and transgenic lines holds much promise in elucidating the metabolic networks and pinpointing the candidate genes underpinning crop stress responses. Additionally, an integrated omics approach encompassing inferences from genomics, transcriptomics, proteomics, and metabolomics will facilitate for cataloging and focusing on key genes for improving key traits of agronomic importance in crops (Kumar et al., 2017).

## Omics Facilitated Crop Improvement for Nutritive Traits

Global climate changes such as increased temperature and elevated CO_2_ levels are associated with decreased nutrient density of some staple crops, ultimately worsening the serious human health challenges suffered by billions of malnourished people in low-income countries (Myers et al., 2014; Macdiarmid and Whybrow, 2019). Moreover, the projected changes could cause reductions in yields of both staple cereal and non-staple legume and vegetable crops, potentially affecting their global availability, affordability and consumption (Scheelbeek et al., 2018; Wang J. et al., 2018; Ray et al., 2019). Since crops are the primary sources of essential nutrients including vitamins, iron (Fe), zinc (Zn), folate, fiber, etc., limited access and consumption of plant-based diets could have serious health implications such as increased risk of non-communicable diseases, and increased nutritional deficiencies that may be difficult to rectify through substitution with other foods (Scheelbeek et al., 2018). In the wake of such climate change scenarios and the need to address human health challenges, improving crop nutritional quality through breeding, agronomic interventions or transgenic approaches become critical. Particularly, enhancing crop micronutrient (particularly Zn, Fe, and vitamins) densities by genetic biofortification through breeding has emerged as a promising, cost-effective and sustainable way to ensure healthy diets to millions of people (Qamar-uz et al., 2017; Garg et al., 2018; Wakeel et al., 2018; Kumar S. et al., 2019).

In order to achieve successful crop nutritional quality improvement, precise identification of major QTLs, genes and metabolic pathways that help interpret the genetic architecture related to plant nutrient acquisition is essential. To this end, several genomic and other omics techniques have been employed to target these nutritive traits, information of which has guided GAB programs (Singh R.K. et al., 2020; Roorkiwal et al., 2021). Major QTLs for nutrition-related traits have been identified in major cereals (reviewed in Singh R.K. et al., 2020) and legumes (reviewed in Roorkiwal et al., 2021). For instance, 14 rice QTLs for cooking and eating quality of grain (including *qTV9* on chr 9) (Park et al., 2019), and 23 rice QTLs for Fe and Zn concentration in grain harboring several candidate genes (including *OsZIP6* on QTL *qZn_5_._1_*.) (Calayugan et al., 2020) were detected. In wheat, five QTLs for gluten strength (including *QGlu.spa-1A* and *QGlu.spa-1B.1* on chr 1A and 1B, respectively) were identified (Ruan et al., 2020). Additionally, 16 wheat QTLs for grain Fe, Zn and protein contents, and 1000-kernel weight were identified, encompassing four Fe QTLs (*QGFe*.*iari-2A*, *QGFe*.*iari-5A*, *QGFe*.*iari-7A* and *QGFe*.*iari-7B*), five Zn QTLs (*QGZn*.*iari-2A*, *QGZn*.*iari-4A*, *QGZn*.*iari-5A*, *QGZn*.*iari-7A* and *QGZn*.*iari-7B*), two protein content QTLs (*QGpc*.*iari-2A* and *QGpc*.*iari-3A*), and five 1000-kernel weight QTLs (*QTkw*.*iari-1A*, *QTkw*.*iari-2A*, *QTkw*.*iari-2B*, *QTkw*.*iari-5B* and *QTkw*.*iari-7A*) (Krishnappa et al., 2017). Besides, 21 QTLs for kernel oil and protein content (including *qOIL08-01*, *qOIL10-01*, *qOIL05-01* and *qOIL06-1* for oil content, and *qPRO01-01*, *qPRO05-01 and qPRO06- 1* for protein content) were identified in maize (Yang Z. et al., 2016). In legumes, QTLs for seed Fe and Zn concentrations in chickpea (Upadhyaya et al., 2016); QTLs affecting seed hardiness in common bean (Sandhu et al., 2018), 8 stable QTLs controlling oil and protein content in soybean (Huang J. et al., 2020), and several QTLs governing oil content, protein content, and fatty acids (linoleic and oleic acids) in groundnut (Sarvamangala et al., 2011; Shasidhar et al., 2017; Roorkiwal et al., 2021) were identified, among others.

In a recent study, using a population of 190 genotypes, Puranik et al. (2020) applied an integration of GBS and GWAS mapping to perform comparative genomics related to identification of genomic regions controlling grain nutrient content (for Fe, Zn, Ca, Mg, K, Na, and protein) in finger millet (*Eleusine coracana* L. Gaertn.). By comparative mapping, they identified several marker-trait associations (MTAs) and predicted associated putative candidate genes underlying significant associations, including *S1_30253617* and probable mitochondrial 3-hydroxyisobutyrate dehydrogenase-like 1 (*LOC101754224*) which were associated with iron content, and SNP S1_5982733 encoding a SEUSS-like transcriptional corepressor which was associated with calcium content (Puranik et al., 2020). Besides, Singhal T. et al. (2021) performed a multi-environment QTL mapping for grain iron and zinc content using bi-parental recombinant inbred lines in pearl millet and identified several QTLs for Fe and Zn, and putative candidate genes within those QTLs involved in Fe and Zn content enhancement. Among the genes identified were *ferritin 1 – chloroplastic*, *potassium transporter 3*, and *aluminum-activated malate transporter 5* (Singhal T. et al., 2021). Considering that pearl millet and other small grains are richly endowed with micro-nutrients and climate-resilience related traits, these candidate QTL regions or genes identified to be linked to such nutritive traits can be targeted for introgression into elite cultivars via GAB (such as marker-assisted backcrossing) or transgenic approaches (Puranik et al., 2020; Roorkiwal et al., 2021). Besides, using multi-omics technologies, cis-regulatory elements (CREs; which are the non-coding DNA containing binding sites for transcriptional factors or other regulatory molecules that influence transcription, Wu et al., 2021) can be precisely identified, analyzed, and targeted for the creation of allelic variation and enhancement of grain quality traits (including grain appearance, milling properties, nutritional value and cooking quality) in crops such as rice via genome editing approaches (Swinnen et al., 2016; Huang L. et al., 2020; Ding et al., 2021).

Meanwhile, maximizing bioavailability of nutrients requires full understanding of the processes involved in crop nutrient uptake, transport, and assimilation into seeds, since multiple genes and complex metabolic pathways are involved. Omics approaches can be applied to help understand the genes and metabolic pathways, including rate limiting steps, involved in nutrient acquisition or biosynthesis, uptake, transport, assimilation and storage processes (Roorkiwal et al., 2021). In particular, manipulating genes and metabolic pathways involved in uptake and transport of Fe, Zn and phosphorus in legumes holds the key for the success of crop nutritional quality improvement. Pathways that can be targeted include beta-carotene biosynthesis, folate biosynthesis, vitamin E biosynthesis and lysine biosynthesis (Kumar A. et al., 2021; Roorkiwal et al., 2021). For instance, metabolomics approaches have been used to target carotenoid biosynthesis pathways (since carotenoids and β-carotene are the primary precursors of vitamin A) and to perform metabolic engineering aimed at increasing β-carotene levels in crops such as rice, maize and potato (reviewed in Sharma V. et al., 2021). Besides, nutritional quality has been improved in maize landraces by enhancing β-carotene content via MABC (Qutub et al., 2021).

Aflatoxin, produced by mostly the fungus *Aspergillus flavus and Aspergillus parasiticus*, is a harmful mycotoxin whose contamination is common in several agricultural crops including groundnut, maize, cotton seed and tree nuts, both pre- and post-harvest (Klich, 2007; Frisvad et al., 2019). Aflatoxin contamination poses serious human and animal health consequences since aflatoxin is carcinogenic, immune-suppressive, cause liver toxicities and abnormalities of physiological development (Kowalska et al., 2017). Fortunately, in groundnut improvement programs, for instance, genomic advances such as sequencing of groundnut diploid progenitors and the cultivated tetraploid groundnut have presented an unparalleled opportunity for enhancing *A. flavus* resistance by helping the decoding of genes and genomic regions underlying host resistance to *A. flavus*. Additionally, metabolomics approaches can be employed to decipher the key metabolic pathways aflatoxin metabolite biosynthesis (reviewed in Ojiewo et al., 2020).

High oleic acid content is a vital quality trait which determines the flavor, stability, shelf-life, and nutritional quality of groundnut and groundnut products. Therefore, the genetic control of this trait is important for high oleic groundnut breeding programs (Amoah et al., 2020). Genetic approaches such as QTL analysis, the use of genetic markers, gene knock-downs and mutants have been successively used to develop high oleic acid (and low linoleic acid) groundnut cultivars, possessing mutated form of *FAD* (fatty acid dehydrogenase) gene (see Ojiewo et al., 2020). Two homologous sequences of the *FAD* gene exist as *FAD2A* and *FAD2B*, owing to the allotetraploid nature of groundnut. These gene homologs are thought to emanate from the two groundnut species genomes, viz., *Arachis ipaensis* and *Arachis duranensis* (Chu et al., 2009; Pandey et al., 2014). The identification of linked allele-specific genetic markers for these two gene homologs has facilitated for breeders to use marker assisted selection and MABC breeding to enhance oleic acid content of elite groundnut varieties (Bera et al., 2018; Desmae et al., 2019). Further, these genomic tools are aiding pyramiding of multiple agronomic traits into a single cultivar (Ojiewo et al., 2020). Going forward, advances in genome sequencing and the availability of diploid and tetraploid genome sequences, as well as the accelerated use of MARS and GS, are envisaged to simplify detection of useful genetic variation, identification of key genes underlying priority traits (such as oleic acid content and low aflatoxin accumulation in groundnut), and introgression of those priority traits into elite cultivars, thereby improving their nutritive value (Desmae et al., 2019).

## Phenomics Facilitated Improvement of Crop Agronomic Traits

Since we have already discussed the recent developments in crop phenotyping methods and tools/technologies in our most recent review (Zenda et al., 2021), here, in this current paper, we will only focus on the application of phenomics to target newly emerging research domains for crop improvement.

### Phenomics Analysis of Root Traits as a New Avenue for Crop Improvement

Root system architecture (RSA) and anatomical traits have important effects on plant function, including acquisition of soil nutrients and water, and subsequent transportation to the aboveground parts (Meister et al., 2014; Paez-Garcia et al., 2015; Zhao et al., 2019). In the past, the lack of information on the measurable genetic or physiological traits has prompted plant breeders to largely focus on optimizing the crop above-ground parts, neglecting the roots. However, the search for new alternative ways to create climate-resilient future crops is making the optimization of both the below-ground and areal plant parts a priority (González and Manavella, 2021). The RSA acts as a major interface between plants and numerous abiotic and biotic stress factors, and helps plants to adapt to these environmental instabilities by sensing and responding to them (Pandey et al., 2017). Such adaptive mechanism or “developmental plasticity” in root growth and development has presented an opportunity for crop breeders to develop climate resilient crops possessing customized RSA that can better adapt to scavenging for diverse supplies of nutrients under specific soil environments (Hodge, 2004; Reynolds et al., 2021).

Several key root structural traits such as primary root length, lateral root length and density, root angle (gravitropism), root tip diameter, crown root number, root hairs, and anatomical root traits (such as root cortical aerenchyma and cell wall modification) can be targeted for QTL mapping and identification of genes underlying these traits under specific abiotic stress conditions (Paez-Garcia et al., 2015; Wasaya et al., 2018). Then, the identified genes can be manipulated via GAB, reverse or forward genetics approaches, or gene editing techniques to develop crops with customized RSA (reviewed in Paez-Garcia et al., 2015). For example, Guo et al. (2020) combined functional phenomics and root economics space analysis approach in winter wheat and identified some root traits, viz., specific root respiration (SRR) and specific root length (SRL), and genomic regions underlying these traits. In particular, they discovered significant variation in SRR and SRL, which were the key aspects of root metabolic and structural costs, respectively. GWASs for the univariate traits identified numerous underlying genetic regions whereas multivariate and PCA-based GWASs offered an enhanced ability to identify the genetics of the root economics space. Moreover, they identified several SNPs linked to these traits that could be used as vital tools for marker-assisted breeding (Guo et al., 2020).

Besides, greater primary root length density enhanced drought tolerance in winter wheat (Djanaguiraman et al., 2019), whilst reduced lateral root branching density but extended length have also improved drought tolerance in maize by enabling access to water available at greater soil depths (Zhan et al., 2015). As an example, we can target such key RSA traits to identify and manipulate genes underlying these traits. Fortunately, the past few years has witnessed massive development of some novel micro-image acquisition techniques and computer based technologies, coupled with several emerging algorithms and softwares that can handle the microscopic images (see Wasaya et al., 2018; Zhao et al., 2019; Demidchik et al., 2020), as well as high-throughput plant phenotyping (HT3P) approaches (reviewed in Li et al., 2021). We can now leverage on these techniques to phenotype the key root traits at cellular, tissue, or organ levels, and these traits can now be estimated from the lab to the field (Tardieu et al., 2017; Li et al., 2021). Ultimately, harnessing and incorporation of these key root traits into crop breeding programs will facilitate for the development of more climate-resilient and efficient crops for the future (Meister et al., 2014; Wasaya et al., 2018).

### Phenomics Applied in Improving Photosynthetic Efficiency and Source-Sink Balance

Photosynthesis process is the basis of plant biomass synthesis or productivity, and the plant photosynthetic machinery is adversely affected by various environmental stressors (reviewed in Muhammad et al., 2021). Therefore, manipulating the photosynthetic processes under environmental fluctuations can be a target for crop improvement (Batista-Silva et al., 2020; González and Manavella, 2021). Phenomics can significantly play a role in accurately detecting plant photosynthetic damages and adaptive response mechanisms under diverse abiotic stress factors, as well predicting the fluctuations in plant biomass or productivity under such environmental conditions (Flood et al., 2016).

Although several bottlenecks in phenotypic evaluation of photosynthesis-related traits have been identified (see Flood et al., 2011), recent advances (and integration) in plant genomics and phenomics technologies have the capability to circumvent these challenges (Furbank and Tester, 2011). Consequently, studying of natural variation (by GWAS analyses) in photosynthesis related traits (including chlorophyll content, chlorophyll reflectance, non-photochemical quenching, photosystem II efficiency, etc.) in diverse crop species under different abiotic stress factors has been made possible (reviewed in van Bezouw et al., 2019). Moving forward, particularly, the investigation of natural variation in photosynthetic efficiency and molecular mechanisms regulating the acclimation of the photosynthetic machinery to these abiotic stresses may be vital in the discovery of novel functional allelic variations, traits and genes that can be targeted for incorporation into current crop improvement programs or used in forward genetic approaches to bio-engineer future crops with enhanced crop photosynthesis efficiency (van Bezouw et al., 2019; Furbank et al., 2020).

It has been long established that photosynthesis flux (source activity) is also dependent on the sink strength (such as grain number and weight in wheat, soybean, rice, etc.). Where an imbalance between source and sink at the whole plant level exists, this can result in reduced expression of photosynthetic genes and accelerated leaf senescence (Paul and Foyer, 2001; Smith et al., 2018). Therefore, the modification of photo-assimilates distribution and accumulation in sink-constrained crops can greatly enhance productivity (Araus et al., 2021). Thus, crop breeders can target increasing mapping and identification of QTLs and genomic regions linked to the rate of grain setting per unit of spike growth at flowering, grain number and grain weight in order to enlarge the sink capacities of crops such as wheat, ultimately improving their photosynthetic efficiencies (Furbank et al., 2020; González and Manavella, 2021; Pretini et al., 2021).

Fortunately, HT3P technologies can facilitate for the analysis of CO_2_ assimilation from the canopy and leaf level (Furbank et al., 2019, 2020). Especially, HT3P data from chlorophyll fluorescence imaging can provide accurate phenotypic dissection of photosynthesis related traits (Dong et al., 2020), and can help to estimate how much biomass (carbon) crops should devote to their root systems in order to fully and efficiently maximize nutrient acquisition with minimal loss of plant fitness and yield (reviewed in Reynolds et al., 2021). Moreover, root anatomical traits such as cell wall remodeling and cortical aerenchyma can also be targeted for phenotyping and genetics analyses since they have shown to significantly limit root respiration, thereby allowing plants to reallocate their biomass in roots or other above-ground plant parts (reviewed in Reynolds et al., 2021). Taken collectively, improving crop photosynthetic efficiency and sink capacity can be targeted for improvement of crop productivity and resilience under future climate conditions, necessitated by improved phenomic and genomics approaches, coupled with gene-editing or bio-engineering technologies.

### Phenomics (Integrated With Multi-Omic Approaches) for Revealing and Exploiting Plant Root-Associated Microbiomes for Improved Crop Health and Climate Resilience

Plant root-associated microbiomes (collection of microbes living inside and around the roots) provide diverse functions that directly influence several plant traits and metabolites are the primary tools plants employ to actively shape their microbiome (De Coninck et al., 2015; Pascale et al., 2020; Chen et al., 2021; Chouhan et al., 2021; Pang et al., 2021). Mechanistically, plant roots exude a cocktail of primary and secondary metabolites which work as growth substrates for some microbial families, exert toxic and antagonistic effects on others, or serve as signals that modulate the plant microbe interactions (Lareen et al., 2016; Chen et al., 2021). Whilst some soil rhizosphere microbial species benefit the plant by acting as growth promoting rhizobacteria or symbionts in enhancing plant pathogen defense and nutrition, some microbes may be commensal or parasitic (reviewed in Lareen et al., 2016; Pascale et al., 2020; Chen et al., 2021). Therefore, dissecting these complex plant - soil rhizosphere - microbiome interactions is critical for designing new approaches for crop resilience to pathogenic and environmental stresses.

Fortunately, the emerging technologies are advancing our understanding of the plant-microbe responses to climate change, as researchers can now investigate host-microbe interactions at a much greater resolution and significance (Dubey et al., 2019b; Pang et al., 2021). In particular, integrated omics approaches, coupled with developments in HTP culturing, synthetic and computational biology, are offering greater insights into the structure and functions of diverse natural microbiomes, and opening a window for creating artificially engineered microbial assemblages aimed at improving crop growth, fitness and resilience to pathogens and numerous abiotic stresses (Trivedi et al., 2021). Combined multi-omics methods are quantifying and revealing the microbiomes features (via HTP amplicon sequencing and metagenomics), microbiomes functions (via metagenomics, metatranscriptomics and metaproteomics), and microbiomes connections with plants and the environment (via metabolomics) (reviewed in Clouse and Wagner, 2021; Trivedi et al., 2021). This has offered new mechanistic insights into how individual or collective microbes underpin plant-microbe interactions for plant health and resilience to climate change (Trivedi et al., 2021). Additionally, plant rhizosphere microbial richness analyses have effectively revealed genotypic and morphological trait variation in crops. For instance, *Phaseolus vulgaris* wild accessions exhibited high relative richness of Bacteroidetes, whilst their counterparts (elite or modern accessions) showed higher abundance of Actinobacteria and Proteobacteria, with the variation being attributed to the plant genotypic and specific root morphological traits (Pérez-Jaramillo et al., 2017; Pascale et al., 2020). Besides, phenomics integrated with bioinformatics, genomic and deep learning approaches are being applied for the diagnosis of crop diseases (reviewed in Adeniji and Babalola, 2020; Marsh et al., 2021).

Moving ahead, plant root-associated microbiomes can be targeted as a source of variation in crop breeding and engineering microbial inoculants to support plant growth and suppress diseases (reviewed in Pascale et al., 2020). Especially, advanced and HTP techniques, such as stable isotope probing, amplicon sequencing, whole-genome shotgun sequencing and metabolomics, coupled with sophisticated bioinformatics softwares and tools (including QIIME, MEGAN, MOTHUR, etc., reviewed in Dubey et al., 2019b; Pang et al., 2021), will become more routinely applied in unlocking the metabolite dialogs between plants and the microbes, and linking those metabolic footprints to key plant genes and phenotypic traits modulating microbiome recruitment or regulation (Chen et al., 2021; Clouse and Wagner, 2021; Trivedi et al., 2021). Taken together, integrating phenomics with other multi-omics approaches provides an invaluable strategy to develop new disease- and climate resilient cultivars via the identification, characterization, manipulation and recruitment of plant rhizospheric microbes into crop breeding and bioengineering programs aimed at improving host plant‘s pathogen resistance and overall fitness and functionality under environmentally challenged conditions.

## Omics Technologies Integrated With Modern Plant Breeding Methods in a Systems Biology Approach for Crop Improvement

Momentous advances in the omics technologies, coupled with reduction in costs for genome sequencing and analysis, as well as developments in bioinformatics tools and databases, have enabled rapid accumulation of huge volumes of omics data that is being routinely used to identify novel alleles and molecular elements underlying key agronomic traits in different crop species. Moreover, these large omics datasets are becoming easily accessible (Chaudhary et al., 2019a). Despite this progress, however, more often, these datasets have been studied independently until recently, and the actual integration of several omics approaches remains tedious due to individualized experimental designs and analytical tools not fit for integrative omics models (Pinu et al., 2019; Pazhamala et al., 2021). Consequently, results from studies employing dis-integrated omics approaches could not provide much insight into the molecular mechanisms regulating key biological systems and complex traits.

Fortunately, integration of multi-omics techniques has emerged as a promising way to address these shortcomings through what is now commonly known as systems biology approach, which is an interdisciplinary research discipline that integrates multi-omics datasets, biological concepts, mathematical models, and machine learning tools to decipher complex biological networks or systems (Pinu et al., 2019). It is premised on multi-omics integration in order to develop a meaningful interpretation of how the genotype is linked to phenotype and subsequent plant responses to environmental stresses (Mohanta et al., 2017). Combining different omics approaches has proven expedient for identifying key candidate genes/proteins and metabolic pathways/networks for functional analysis and/or elucidation of complex molecular underpinnings to several important agronomic traits or plant abiotic and biotic stress responses. For instance, integrated transcriptomics, proteomics and metabolomics analyses of the mechanisms regulating low tiller production in low-tillering wheat identified 474, 166, and 28 tillering-associated differentially expressed genes, proteins, and 28 metabolites, respectively (Wang Z. et al., 2019). Comprehensive metabolic pathway enrichment analyses of these genes, proteins and metabolites pinpointed to three TF families (*GRAS*, *GRF*, and *REV*) and lignin biosynthesis pathway as responsible for the inhibition of tiller development in low-tillering wheat cultivars (Wang Z. et al., 2019). Besides, conjoint analysis (coupling comparative cytology with transcriptomic and metabolomic approaches) to understand the mechanisms underlying *Solanum nigrum* L. response to cadmium toxicity revealed key differentially expressed genes and metabolites, including laccase, peroxidase, D-fructose, and cellobiose, that were associated with cell wall biosynthesis, implying that the cell wall biosynthesis pathway plays a central role in Cd detoxification in *Solanum nigrum* (Wang et al., 2022). Combined transcriptomic and metabolomic approaches applied in maize to analyze gene regulatory networks modulating *Rp1-D21* mutant-mediated hypersensitive pathogen defense response revealed that four uridinediphosphate-dependent glycosyltransferase (UGT) (*ZmUGTs)* genes were highly expressed, whilst the SA biosynthesis and phenylpropanoid biosynthesis pathways were induced at both the transcriptional and metabolic levels, suggesting that *ZmUGT* genes may be involved in maize defense response by regulating SA homeostasis (Ge et al., 2021). Earlier, the epigenetic-based amalgamation of multi-omics approaches elucidated the critical role DNA methylation and play in lipid biosynthesis regulation and spatio-temporal modulation of ROS during cotton fiber development (Wang et al., 2016). Thus, integrated omics approaches facilitate for in-depth understanding of complex physiological and molecular mechanisms underpinning several key traits of agronomic importance (Singh N. et al., 2020), as well as formulation of predictive models of those key traits using large molecular datasets (Scossa et al., 2021). This all-encompassing approach is crucial and a very promising strategy for creating climate-smart crop cultivars (Chaudhary et al., 2019a, b; Jha et al., 2020; Pazhamala et al., 2021; Raza et al., 2021b).

**FIGURE 2:**
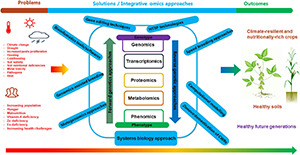
Abstract illustration of the role of integrated omics approaches in anchoring the development of climate-smart future crops. Integrated multi-omics strategies coupled with forward and reverse genetics methods, as well as advanced plant breeding, gene editing, mutagenomics and computational modeling techniques in a systems biology approach facilitate for the creation of climate-resilient and nutrition-rich crops. HT3P, high-throughput plant phenotyping platforms; CWRs, crop wild relatives.

Meanwhile, these multi-omics generated data will need to be integrated with modern plant breeding and gene editing technologies in order to provide a comprehensive, time- and cost-effective strategy for targeting candidate genes regulating key agronomic and nutrition-related traits essential for developing climate-ready crops (Liu H.J. et al., 2020; Gogolev et al., 2021; Kumar R. et al., 2021). Such modern plant breeding technologies include double-haploid (DH) breeding (Yan et al., 2017), induced mutagenesis (Kharkwal and Shu, 2009), CRISPR-Cas based gene editing technologies (see Ahmar et al., 2020; Steinwand and Ronald, 2020; Fiaz et al., 2021; Gao, 2021; Kumar A. et al., 2021; Marsh et al., 2021; Sinha et al., 2021), and the single seed chipping (SSC) facilitated marker-based early generation selection (MEGS) technique (Parmar et al., 2021), among others. For instance, the SSC facilitated MEGS protocol could be used to successfully advance 3.5 breeding generations in groundnuts, and could significantly cut the time required to complete the entire breeding cycle by approximately 6-8 months. Additionally, the SSC technique did not significantly affect germination percentage (as it remained high, 95-99%) (Parmar et al., 2021). Therefore, this technique could be an indispensible tool to promote high-throughput genotyping and speed breeding of climate-smart groundnut (and possibly other legume) crop cultivars. Further, improved crop management practices that help maintain stabilized yields under resource constrained environments, including conservation agriculture and the use of melatonin to enhance crop stress tolerance will remain more relevant (Fahad et al., 2017; Zenda et al., 2020). This holistic approach to crop improvement for resilience to climate change and higher nutritive value is summarized in Figure 2.

## Conclusion and Future Prospects

Here, we have cited several relevant examples to highlight how various omics approaches have anchored the crop improvement programs. Deployment of these omics techniques, particularly genomics, transcriptomics, proteomics, metabolomics and phenomics to study plant responses to numerous abiotic and biotic stresses has been vital in revealing several key genes, proteins and metabolic pathways underlying several quantitative and quality traits of agronomic importance in major crop species. Some of the identified candidate genes and metabolic pathways have been deployed in genomics-assisted or marker assisted breeding programs via molecular breeding approaches or genetic-engineering methodologies. Moreover, the recent advances in metabolomics and high-throughput phenotyping platforms have fortified the utility of genomics, transcriptomics and proteomics. Particularly, metabolomics is currently receiving special attention, owing to the role metabolites play as metabolic intermediates and close links to the phenotypic expression. Additionally, high throughput phenomics applications are driving the targeting of new research domains such as root system architecture analysis, and exploration of plant root-associated microbes for improved crop health and climate resilience. Further, single-cell transcriptomics and ionomics have emerged as the new “kids on the block” showing great promise for effective use in solving complex biological questions in the near future, although several technical and experimental design related challenges still need to be resolved. Fortunately other areas such as gene editing, bioinformatics analysis tools and softwares, and machine learning have also witnessed significant progress to support the advances in omics techniques. Leveraging on these developments, we envisage that combining multi-omics methods with modern plant breeding techniques, HTP experimental techniques, advanced bioinformatics, and computational modeling tools in a systems biology approach will facilitate for the development of sustainably higher yielding and nutritionally rich climate-resilient crops for the future.

## Author Contributions

TZ and HD conceived the idea. TZ, SL, AD, JL, YW, XL, and NW performed the literature search. TZ prepared and wrote the original draft manuscript, and designed the figures. TZ, SL, AD, JL, YW, XL, NW, and HD reviewed and edited the manuscript. TZ and SL prepared the tables. HD involved in funding acquisition. All authors have read and agreed to the published version of the manuscript.

## Conflict of Interest

The authors declare that the research was conducted in the absence of any commercial or financial relationships that could be construed as a potential conflict of interest.

## Publisher’s Note

All claims expressed in this article are solely those of the authors and do not necessarily represent those of their affiliated organizations, or those of the publisher, the editors and the reviewers. Any product that may be evaluated in this article, or claim that may be made by its manufacturer, is not guaranteed or endorsed by the publisher.
